# Bacterial Diversity and Biogeochemistry of Two Marine Shallow-Water Hydrothermal Systems off Dominica (Lesser Antilles)

**DOI:** 10.3389/fmicb.2017.02400

**Published:** 2017-12-04

**Authors:** Petra Pop Ristova, Thomas Pichler, Michael W. Friedrich, Solveig I. Bühring

**Affiliations:** ^1^Hydrothermal Geomicrobiology Group, MARUM - Centre for Marine Environmental Sciences, University of Bremen, Bremen, Germany; ^2^Geochemistry and Hydrogeology, University of Bremen, Bremen, Germany; ^3^Microbial Ecophysiology Group, Faculty of Biology/Chemistry, Centre for Marine Environmental Sciences, University of Bremen, Bremen, Germany

**Keywords:** shallow-water, hydrothermal systems, bacterial diversity, geochemistry, Dominica

## Abstract

Shallow-water hydrothermal systems represent extreme environments with unique biogeochemistry and high biological productivity, at which autotrophic microorganisms use both light and chemical energy for the production of biomass. Microbial communities of these ecosystems are metabolically diverse and possess the capacity to transform a large range of chemical compounds. Yet, little is known about their diversity or factors shaping their structure or how they compare to coastal sediments not impacted by hydrothermalism. To this end, we have used automated ribosomal intergenic spacer analysis (ARISA) and high-throughput Illumina sequencing combined with porewater geochemical analysis to investigate microbial communities along geochemical gradients in two shallow-water hydrothermal systems off the island of Dominica (Lesser Antilles). At both sites, venting of hydrothermal fluids substantially altered the porewater geochemistry by enriching it with silica, iron and dissolved inorganic carbon, resulting in island-like habitats with distinct biogeochemistry. The magnitude of fluid flow and difference in sediment grain size, which impedes mixing of the fluids with seawater, were correlated with the observed differences in the porewater geochemistry between the two sites. Concomitantly, individual sites harbored microbial communities with a significantly different community structure. These differences could be statistically linked to variations in the porewater geochemistry and the hydrothermal fluids. The two shallow-water hydrothermal systems of Dominica harbored bacterial communities with high taxonomical and metabolic diversity, predominated by heterotrophic microorganisms associated with the Gammaproteobacterial genera *Pseudomonas* and *Pseudoalteromonas*, indicating the importance of heterotrophic processes. Overall, this study shows that shallow-water hydrothermal systems contribute substantially to the biogeochemical heterogeneity and bacterial diversity of coastal sediments.

## Introduction

Hydrothermal systems represent extreme environments in the ocean, where reduced and often acidic fluids of high temperature, laden with heavy and toxic metals exit the seafloor. Hydrothermal systems generally exhibit two types of fluid discharge: (1) focused discharge from discrete hydrothermal vents and (2) dispersed or diffuse discharge of fluids (Pichler and Veizer, 1999; Price et al., 2007; Dekov et al., 2010). Diffuse flow hydrothermal systems are characterized by low temperature fluids (<0.2 to ~100°C), composed of mixtures of cold seawater and magmatically heated fluids, slowly discharging through sulfide mounds, fractured lava flows, bare sediments and assemblages of bacterial mats and macrofauna (Bemis et al., 2012). Particularly diffuse venting through sediments in hydrothermal areas creates unique environments with well-developed vertical and lateral (physico)geochemical gradients at scales ranging from a few millimeters to several meters, i.e., with steep and gentle slopes (Price et al., 2007; Karlen et al., 2010). In the uppermost sediment layers, mixing of ascending hydrothermal fluids rich in electron-donors with cold ambient seawater full of electron-acceptors, provides chemical energy for microorganisms (Amend and Shock, 1998). Moreover, due to the mixing of these two water bodies, dissolved compounds precipitate and form energy-rich solid surfaces that can be exploited by microorganisms (e.g., Holden et al., 2012). Microorganisms of hydrothermal ecosystems have the capacity to transform a large range of chemical compounds derived from hydrothermalism through different redox coupling reactions (Fisher et al., 2007; Jørgensen and Boetius, 2007), and to fix carbon via chemosynthesis (Sievert and Vetriani, 2012). These microbial communities possess diverse metabolisms and are capable of oxidizing, e.g., H_2_, H_2_S, CH_4_, Fe^2+^, NH_3_ and Mn^2+^ using variety of electron acceptors, e.g., O_2_, ${\text{NO}}_{3}^{-}$, ${\text{SO}}_{4}^{2-}$, Fe^3+^ (Fisher et al., 2007; Kato et al., 2012). This promotes the formation of hydrothermal ecosystems with high productivity and biomass, as well as specialized biodiversity (Jørgensen and Boetius, 2007).

Hydrothermal ecosystems have a global distribution from the shallow-water coastal regions to the abyssal plain, occurring on divergent boundaries at mid-ocean ridge systems and convergent boundaries associated with seamounts and island arc volcanoes (Tarasov et al., 2005; Price and Giovannelli, 2017). Shallow-water systems differ from the well-known deep-sea systems in that they lack the emblematic deep-sea vent megafauna and are situated within the photic zone, at water depths shallower than ~200 m, where light still reaches the seafloor (Tarasov et al., 2005). Therefore, in addition to chemosynthetic processes, microbial communities of shallow-water systems may also use light for the production of biomass via photosynthesis, resulting in high biological productivity and likely even more complex microbial communities relative to the deep-sea counterparts (Wang et al., 2015). Despite this, little is known about the ecology of microbial communities of shallow-water hydrothermal systems and they remain largely understudied compared to their deep-sea counterparts (Dawson et al., 2016; Miranda et al., 2016; Gomez-Saez et al., 2017).

*Archaea* and *Bacteria* are important members of the communities at shallow-water hydrothermal systems, because they provide the bases of the food webs at these localities and are responsible for the transformation of compounds transported by the venting fluids (Sievert et al., 2000; Lentini et al., 2014). Archaea dominate the hottest areas at hydrothermal vents, while the relative contribution of bacteria is higher in the areas with lower temperatures (Rothschild and Mancinelli, 2001; Price and Giovannelli, 2017). Studies on shallow-water hydrothermal systems using high-throughput sequencing are still few (Price and Giovannelli, 2017 and references therein), and thus the ecology and in specific the extent of microbial diversity in these ecosystems remains poorly understood. Metabolically and taxonomically diverse bacteria dominate individual shallow-water hydrothermal systems, suggesting a high degree of heterogeneity among these ecosystems (Gugliandolo et al., 2012; Zhang et al., 2012; Giovannelli et al., 2013; Lentini et al., 2014; Price and Giovannelli, 2017 and references therein). The factors, however, selecting for the different bacterial communities and thus promoting high heterogeneity remain elusive. The overall aim of this study was to contribute knowledge about the microbial ecology of shallow-water hydrothermal ecosystems, and to assess their diversity and the biogeochemical factors shaping the communities in these ecosystems.

We investigated sediments from two marine shallow-water hydrothermal systems off the island of Dominica (Lesser Antilles). Given the relatively moderate temperatures of the Dominica shallow-water hydrothermal systems (55 and 75°C), the main focus of this study was on the bacterial part of the microbial communities. Our study is the first report on the natural bacterial communities not only of Dominica, but also for the whole Lesser Antilles arc. Here, we combined fingerprinting Automated Ribosomal Intergenic Spacer Analysis (ARISA) and Illumina sequencing techniques with porewater geochemistry analyses to gain understanding of the diversity of the bacterial communities in the context of their biogeochemical surroundings. For comparison, in addition we sampled two areas that were not impacted by fluid venting. The main questions addressed here are: (i) how do the biogeochemistry and associated bacterial communities of shallow-water hydrothermal sediments compare to those not impacted by hydrothermal fluids, (ii) which factors shape the bacterial diversity of hydrothermally-influenced sediments, and (iii) do individual shallow-water hydrothermal systems promote different bacterial diversity.

## Materials and methods

### Description of sampling sites

**Figure 1:**
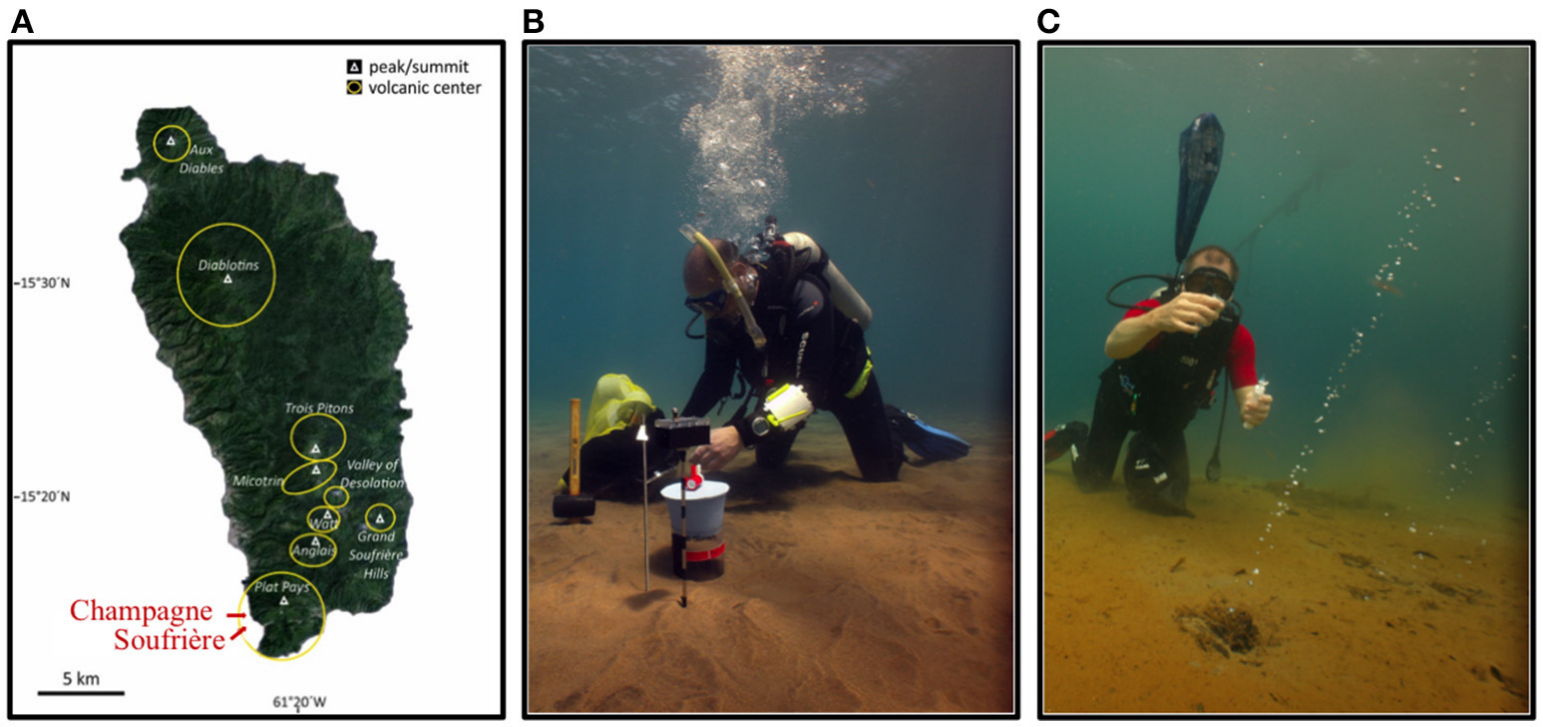
**(A)** Map of Dominica Island displaying the two investigated areas (modified after Gomez-Saez et al., 2017). **(B,C)** *In situ* photos depicting the sediment conditions at Soufrière and Champagne hydrothermal sites. Orange to red color of the seafloor is due to the Fe-precipitates that overly the sediment at both sites. Photos courtesy of A. Madisetti (April 2013).

In April 2013, two shallow-water hydrothermal areas (5 m water depth) in the southwest of Dominica Island, i.e., Champagne Hot Springs (CHS_HT_; N 15.245846, W 61.373125) and Soufrière Bay (SOU_HT_; N 15.232771, W 61.362047) were investigated (Figure 1A). Dominica is part of the volcanically active Lesser Antilles arc system and is uniquely characterized by a volcanic complex with potentially nine active centers, both terrestrial and marine (Lindsay et al., 2005; Joseph et al., 2011). Hydrothermally-impacted sampling sites were visually identified via SCUBA diving, through the observation of gas bubbles, discharge of hydrothermal fluid from discrete vents and diffusive venting through the surrounding sediments. Hydrothermal sites were located in the vicinity of local villages and a substantial anthropogenic influence could be visually identified at both sites. The sediments in the vicinity of hydrothermal venting at both sampling sites were covered by a layer of reddish hydrous ferric oxide precipitates (Figures 1B,C; see also McCarthy et al., 2005). Visually, Soufrière appeared to be more active, due to stronger gas bubbling. SCUBA diving surveys revealed that the hydrothermal activity at the Champagne site seemed to have declined compared to previous years (Pichler, unpublished observations). The fluids at the two sites were characterized by elevated temperatures (55–75°C) and Fe^2+^ concentrations (5–13 mg L^−1^), as well as decreased pH (6.3) and salinity (11–17‰) compared to seawater (Table 1) (Kleint et al., 2015, 2017).

**Table 1:** Geochemical characteristics of hydrothermal fluids and bottom water at the Champagne and Soufrière hydrothermal sites (HT) compared to their respective background samples (BG) and seawater.

**Location**	**Sample type**	**pH**	**Salinity (‰)**	**Fe^2+^ (mg L^−1^)**	**Fe total (mg L^−1^)**	**T (°C)**	**O_2_ (%)**
CHS_BG_	Bottom water	7.57	33	0.9	1.8	29.5	n.d.
CHS_HT_	Bottom water	6.4	14.3	6–7	6–7	46	n.d.
CHS_HT_	Hydrothermal fluids	6.3	17.3	5–6	5–6	75	n.d.
SOU_BG_	Bottom water	7.95	32.5	<0.1	<0.1	29.5	70
SOU_HT_	Bottom water	6.38	12.8	10.5	10.5	55	65.1
SOU_HT_	Hydrothermal fluids	6.33	10.9	13	13	55	60.6
Dominica	Seawater	7.9	34	b.d.	b.d.	n.d	n.d.

*n.d., not determined; b.d., below detection limit*.

For comparison, we investigated background sediments (CHS_BG_ and SOU_BG_) from both sampling sites that were not impacted by hydrothermalism upon the sampling.

Prior to sampling, temperature measurements were performed at all sampling sites (5 cm resolution, max. 25 cm depth), using a custom-made underwater electric temperature probe. Sediments were retrieved by SCUBA divers using hand operated polycarbonate push cores (∅ 7.5 cm, length 30 cm). Immediately after recovery, the sediments were extruded from the push cores, incrementally sliced in 2-cm resolution using sterile equipment, and subsampled for different types of analyses.

### Sampling and geochemical analyses

At both sampling locations, Soufrière and Champagne, we sampled hydrothermal fluid from discrete vents, bottom seawater and porewater from hydrothermally-impacted sediments. Hydrothermal areas were identified by the elevated sediment temperature compared to the background, emergence of gas bubbles and reddish precipitates of ferric oxide. To serve as background data for comparison, we also collected porewater and seawater away from the area of hydrothermal activity. The bottom water samples were a mixture of hydrothermal fluid and seawater collected just above the sediment-seawater interface. The bottom water and hydrothermal fluids were collected into 60 mL syringes. Temperature of fluids was measured *in situ* with a custom-made temperature probe, while pH, salinity and dissolved oxygen were measured on shore in sample aliquots using a WTW® multimeter (Weilheim, Germany). Porewater was collected from sediment cores and extracted in 2-cm steps (max. 20 cm sediment depth), using Rhizon moisture samplers with a pore size 0.1 μm (Rhizosphere Research Products, Wageningen, Netherlands) (Seeberg-Elverfeldt et al., 2005), which were inserted into holes of the push core liners. Samples were immediately separated into aliquots for the determination of total sulfide (totS^2−^), dissolved organic carbon (DOC), dissolved inorganic carbon (DIC), anion and cation concentrations. Total sulfide was determined spectrophotometrically (Pharo 100 Spectroquant, Merck KGaA, Darmstadt, Germany) on subsamples that were fixed with 5% zinc acetate (Cline, 1969). DOC subsamples were pre-filtered with 0.2 μm PES filters, acidified with suprapure HCl to pH = 2 and stored in sealed precombusted glass vials in the field. In the laboratory, DOC concentration was determined on a TOC analyzer (multi N/C® 2100S, Analytik Jena, Germany). Subsamples for total iron (Fe^T^), potassium (K^+^), magnesium (Mg^2+^), total manganese (Mn^T^), boron (H_3_${\text{BO}}_{4}^{◦}$), calcium (Ca^2+^), sodium (Na^+^), strontium (Sr^2+^) and silica (H_4_${\text{SiO}}_{4}^{◦}$) were acidified to 1% with ultrapure HNO_3_ in the field, and stored at 4°C until measured by inductively coupled-optical emission spectrometry (Optima 7300 ICP-OES; Perkin-Elmer, Rodgau, Germany) following established procedures (Pichler et al., 2004). Measurements of anion concentrations, i.e., chloride (Cl^−^) and sulfate (${\text{SO}}_{4}^{2-}$) were performed by ion chromatography (Metrohm 883 IC Basic Plus; Metrohm AG, Herisau, Switzerland) on previously frozen (−20°C) subsamples. All geochemical data is archived in PANGAEA, Data Publisher for Earth & Environmental Science and is publically available (Pop Ristova et al., 2017b).

Samples used for determination of sediment grain sizes were frozen in the field (−20°C). In the laboratory, sediment grain sizes were determined by sieving of dried sediments with different mesh sizes i.e., 0.5, 0.25, 0.125, and 0.063 mm.

### Bacterial community analyses

Analysis of the bacterial community was performed on the same sediment cores used for geochemical analysis. Sediments were sliced in 2-cm resolution down to a maximum depth of 20 cm. Sediment samples were preserved in a DNAse- and RNAse-free LifeGuard® Soil Preservation Solution (MoBio Laboratories, Inc., Carlsbad, CA, USA) and stored at −20°C till further processing in the home laboratory.

DNA was extracted from 0.5 g of sediment using the FastDNA®SPIN Kit for Soil (MP Biomedicals, Irvine, USA), following manufacture's protocol with small modifications. Modifications of the protocol included two additional steps: (i) incubation of samples in a water bath at 65°C for 10 min, after protocol's step four of homogenization, and (ii) incubation of samples in water bath at 37°C for 15 min, during protocol's step 16 of final DNA elution. DNA was finally eluted in 50 μl 1 × Tris-EDTA buffer (Promega, Madison, WI, USA).

Changes in the bacterial community were assessed with two molecular techniques, i.e., Illumina sequencing and Automated Ribosomal Intergenic Spacer Analysis (ARISA). Standardized amounts of DNA (10 ng) from each sample were amplified in triplicates using the ARISA-specific ITSReub and ITSF (Hex-labeled) primers (Fisher and Triplett, 1999; Cardinale et al., 2004). The PCR procedure, cleaning of PCR products and separation of amplified fragments via capillary electrophoresis were done as described previously (Ramette, 2009), with the exception that here we used PeqLab dNTPs and peqGOLD Taq DNA Polymerase (PeqLab Biotechnology, Erlangen, Germany). Fragments between 100 and 1,000 bp length were binned into ARISA operational taxonomic units (OTUs; 2 bp window frame) using customized scripts, publically available at https://www.mpi-bremen.de/en/Software-4.html#section1549. PCR triplicates were merged by retaining all fragments present in at least two of the three replicates.

A selection of samples from each site, encompassing the following depth intervals 0–2 cm, 2–4 cm, 8–10 cm, 16–18 cm, were analyzed by Illumina MiSeq sequencing technology. The V3–V4 hypervariable region of the 16S rRNA gene was sequenced using the S-D-Bact-0341-b-S-17 (5′-CCTACGGGNGGCWGCAG-3′) and S-D-Bact-0785-a-A-21 (5′-GACTACHVGGGTATCTAATCC-3′) bacterial primers (Klindworth et al., 2013). Sequencing was performed with the Illumina MiSeq (pair-end technology) at MR DNA laboratory (Molecular Research LP, Shallowater, TX, US). Multifasta files were parsed, checked for quality, and trimmed with the split_libraries.py command as implemented in QIIME v1.9.1 (Caporaso et al., 2010). Processing of raw sequences, including alignment, quality control, dereplication, clustering and classification, was done with the SILVAngs analysis pipeline 1.2 (SILVA SSU Ref dataset 119.1; Quast et al., 2013). EzBioCloud was used to assign pairwise similarity to the most abundant OTUs against their closest (un)cultured relatives (Yoon et al., 2017). Operational taxonomic units (OTU_0.03_) were formed by clustering the sequences at 97% sequence's identity level. Sequence fastq files were deposited at the GenBank Sequence Read Archives (http://www.ncbi.nlm.nih.gov), and are publically available under the BioProject ID: PRJNA386676.

All analyses, except alpha diversity calculations, were done on the dataset with removed singleton sequences, i.e., OTU_0.03_ represented by only one sequence within the whole dataset. OTUs that were classified only to the taxonomical level of family, as well as those matching chloroplast and mitochondrial sequences were excluded from the analysis.

### Statistical analysis

Differences between sampling sites, based on their geochemistry or bacterial community structure, were determined and visualized with Principal Component Analysis (PCA; based on Euclidean distance) and nonmetric multidimensional scaling analysis (NMDS; based on Bray-Curtis distance), respectively. Separation of groups identified on the NMDS was tested for significance using the non-parametric Analysis of Similarity (ANOSIM) test. Individual and combined effects of the measured geochemical variables on the structure of microbial communities were assessed by redundancy analysis (RDA) and canonical variation partitioning. Priorly individual geochemistry variables were checked for co-linearity (Supplementary Table 1). Most of the geochemical parameters were significantly correlated with H_4_${\text{SiO}}_{4}^{◦}$. Silica was thus selected and kept in the RDA, as this parameter represents a good proxy for the influence of hydrothermalism. Prior to the RDA the geochemical data were normalized (logarithmic transformation) and standardized (z-score transformation), while the bacterial dataset was Hellinger transformed, as recommended for these types of datasets (Ramette, 2007). All analyses of the beta-diversity, i.e., differences in the microbial community structure between sites, were done on the ARISA-based dataset.

Alpha diversity (i.e., OTU_0.03_ richness) of each of the samples was determined by calculating the Chao1, ACE and Shannon diversity indices, based on the Illumina sequences dataset. The sequence dataset was previously normalized to the sample with the smallest number of sequences (CHS_HT__8–10 cm, 33479; Table 2). Difference in alpha diversity between hydrothermal and background samples was tested for significance using the Kruskal Wallis and Mann Whitney *U* test for unmatched samples. All statistical analyses were performed with the R software, using vegan (Oksanen et al., 2015) and ggplot2 (Wickham, 2009) packages, as well as custom-based scripts.

**Table 2:** Sequence data characteristics and estimated OTU_0.03_ richness indices of bacterial samples.

	**Number of sequences**	**Number of OTU_0.03_**	**Number of singleton OTU_0.03_**	**Percentage of singleton OTU_0.03_**	**Number of OTU_0.03_ w/o singletons**	**Shannon diversity**	**Chao1**	**ACE**
CHS_BG__0-2cm	149,507	26,813	19,251	71.8	7,562	7.6	30,195	30,095
CHS_BG__2-4cm	308,097	39,072	27,334	70	11,738	7.5	23,286	22,094
CHS_BG__8-10cm	69,604	12,638	8,991	71.1	3,647	7	26,223	26,328
CHS_BG__16-18cm	56,168	22,418	16,440	73.3	5,978	8.9	55,989	58,295
CHS_HT__0-2cm	183,699	22,231	15,597	70.2	6,634	6.7	21,919	21,388
CHS_HT__2-4cm	82,235	24,699	18,222	73.8	6,477	8.4	48,864	49,983
CHS_HT__8-10cm	33,479	5,004	3,627	72.5	1377	6	18,182	18,763
CHS_HT__16-18cm	190,484	22,104	15,392	69.6	6,712	6.7	20,397	21,228
SOU_BG__0-2cm	360,324	45,052	31,774	70.5	13,278	7.7	25,301	24,317
SOU_BG__2-4cm	52,594	13,629	10,001	73.4	3,628	7.8	37,660	37,483
SOU_BG__8-10cm	72,765	24,101	17,539	72.8	6,562	8.8	49,965	48,931
SOU_BG__16-18cm	88,587	26,753	19,252	72	7,501	8.7	45,670	45,754
SOU_HT__0-2cm	235,478	28,446	20,148	70.8	8,298	6.9	22,578	22,549
SOU_HT__2-4cm	127,220	19,706	13,903	70.6	5,803	7.3	25,693	26,057
SOU_HT__8-10cm	167,454	24,973	17,715	70.9	7,258	6.8	24,837	25,386
SOU_HT__16-18cm	36,142	11,114	8,080	72.7	3,034	8	39,709	39,392

## Results

### Comparison of geochemistry of hydrothermal and background sites

The bottom waters at the background sites were similar to the seawater with a temperature of 29.5°C, a salinity of 33‰ and a pH between 7.6 and 7.9 (Table 1). In contrast, bottom water at the hydrothermal sites showed a clear hydrothermal impact, with decreased pH (6.4) and salinity (13‰ and 14‰) as well as elevated temperatures (46°C and 55°C). The Fe^T^ concentrations were elevated at the venting sites (6–11 mg L^−1^), but hardly detectable in the background samples.

**Figure 2:**
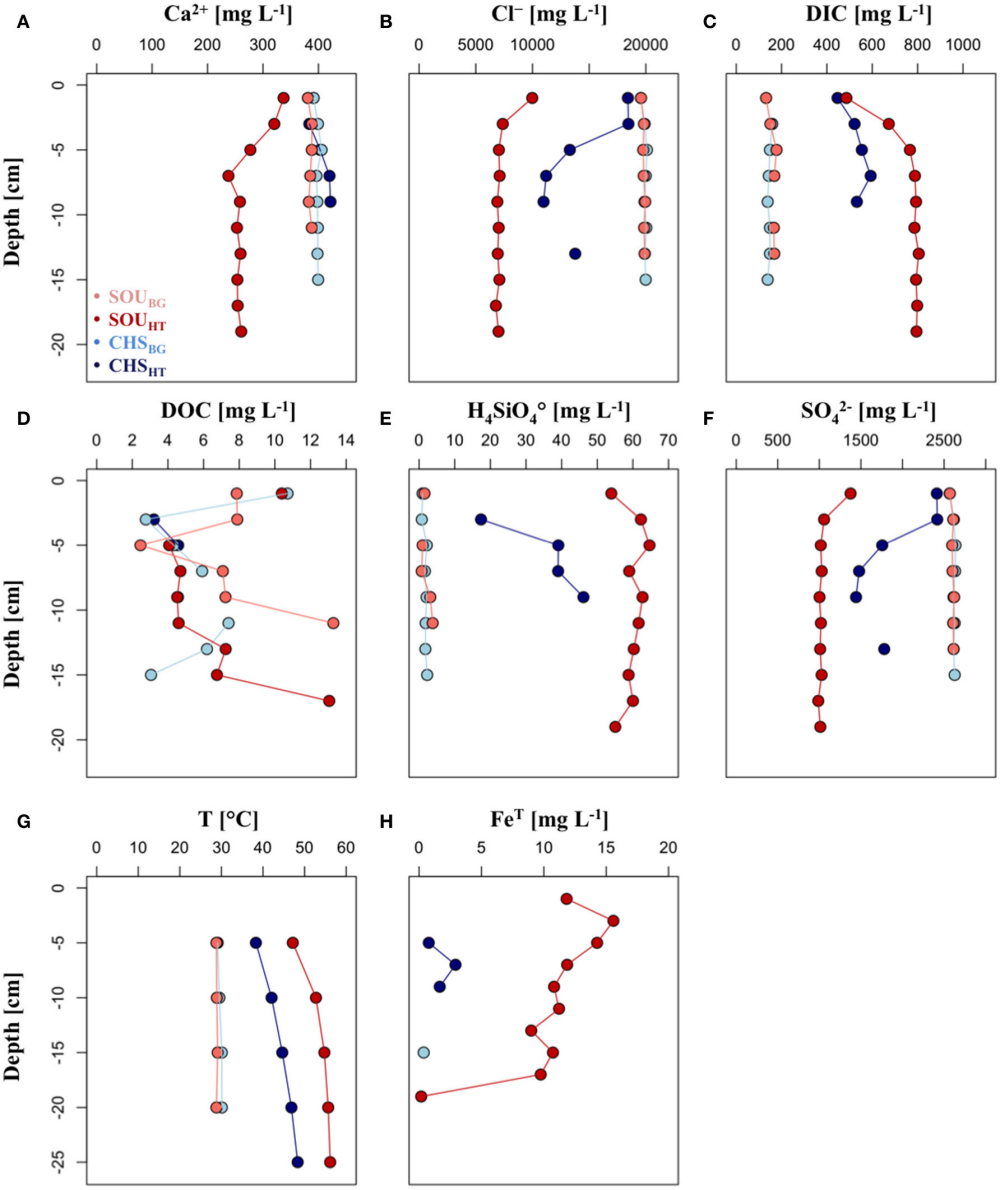
Depth profiles of **(A)** calcium, **(B)** chloride, **(C)** dissolved inorganic matter, **(D)** dissolved organic matter, **(E)** silica, **(F)** sulfate, **(G)** temperature, and **(H)** iron.

Principal component analysis showed that the porewater in the area of venting had a substantially different chemical composition compared to the background sites (Supplementary Figure 1). This difference was slightly greater at the SOU_HT_ than at the CHS_HT_ site. Sediment porewaters from the area of venting had increased concentrations of H_3_${\text{BO}}_{4}^{◦}$, Fe^T^, H_4_${\text{SiO}}_{4}^{◦}$ and DIC, as well as decreased levels of ${\text{SO}}_{4}^{2}$, Cl^−^, Na^+^, K^+^, and Mg^2+^ compared to their respective background sites (Figure 2, Supplementary Figure 2). Sulfide was not detected in the porewater at any site, although small amounts of dissolved totS^2−^ were detected in the hydrothermal fluids (0.44 mg L^−1^).

### Geochemistry of different shallow-water hydrothermal systems

Most of the investigated geochemical parameters at both sites were linearly related with seawater and the hydrothermal fluid from the on-land hot springs, i.e., Sulfur Springs (McCarthy et al., 2005) (Supplementary Figure 3). Similar to McCarthy et al. (2005) we used seawater and the terrestrial Sulfur Spring fluids as respective end-members. The intermediate position of the SOU_HT_ and CHS_HT_ porewater and hydrothermal fluids between the two end-members (Supplementary Figure 3) indicates that mixing is the main control for their chemical composition. The only exceptions were Ca^2+^, ${\text{SO}}_{4}^{2-}$ and H_3_${\text{BO}}_{4}^{◦}$ at CHS_HT_ and ${\text{SO}}_{4}^{2-}$ and H_3_${\text{BO}}_{4}^{◦}$ at SOU_HT_ (Supplementary Figure 3).

Based on all measured parameters the two hydrothermal sites had a substantially different hydrothermal fluid and porewater geochemistry (Figure 2, Supplementary Figure 1). Temperature rapidly increased with sediment depth at both hydrothermal sites, and reached maximum values in the deepest investigated layer (56.2 ± 0.4°C at SOU_HT_ and 48.3 ± 0.5°C at CHS_HT_ at 25 cm; Figure 2). On average the temperature at SOU_HT_ was 9°C higher than at CHS_HT_. Showing highly similar depth patterns, Cl^−^ and ${\text{SO}}_{4}^{2-}$ concentrations were low over the entire investigated depth at CHS_HT_ (Cl^−^ = 7,322 ± 946 mg L^−1^ and ${\text{SO}}_{4}^{2-}$ = 1,053 ± 115 mg L^−1^), while at SOU_HT_ they dropped from 18,422 mg L^−1^ and 2,413 mg L^−1^ in the surface to 10,975 mg L^−1^ and 1,441 mg L^−1^ at 8–10 cm depth, respectively. Silica was on average two times more concentrated in the porewater of SOU_HT_ (60 ± 3 mg L^−1^) than at CHS_HT_ (35 ± 12 mg L^−1^). SOU_HT_ had also higher DIC concentration (max. 805 mg L^−1^) compared to CHS_HT_ (max. 593 mg L^−1^), nevertheless both sites showed similar pattern of rapid DIC increase in the top 8 cm of the sediment.

Iron could be detected in only a few samples at the CHS_HT_, where it remained below 3 mg L^−1^ (Figure 2), while almost five times higher concentrations were measured throughout the whole investigated depth at the SOU_HT_ (max. 16 mg L^−1^). Calcium concentrations decreased in the surface layers at the SOU_HT_ (337 to 238 mg L^−1^) and they were lower over the entire investigated depth compared to CHS_HT_ (407 ± 18 mg L^−1^), which in turn had similar values as the background samples. Dissolved organic carbon (3–13 mg L^−1^) showed no clear pattern with sediment depth and it did not differ between the investigated sites.

The grain size of the sediments at SOU_HT_ was smallest (0.07 mm), and about half the size compared to the other sites (Supplementary Figure 4).

### Diversity and community structure of bacterial communities

Differences in the alpha diversity (estimated OTU_0.03_ richness) between individual samples, based on all calculated species estimated indices were large. Lowest Chao1 of 18,182 was detected for CHS_HT__8–10 cm and almost three times higher for the SOU_BG__8–10 cm sample (Chao1 = 49,965; Table 2). At both hydrothermal sites alpha diversity did not vary with sediment depth, and all hydrothermal samples had in general lower estimated species diversity compared to the respective background sediments (Chao1 = 36,786 ± 12,486 at background sites and Chao1 = 27,772 ± 10,743 at the hydrothermal sites). Differences in the alpha diversity between hydrothermal and background sites were statistically significant (Wilcox test *W* = 64, *p* < 0.001).

**Figure 3:**
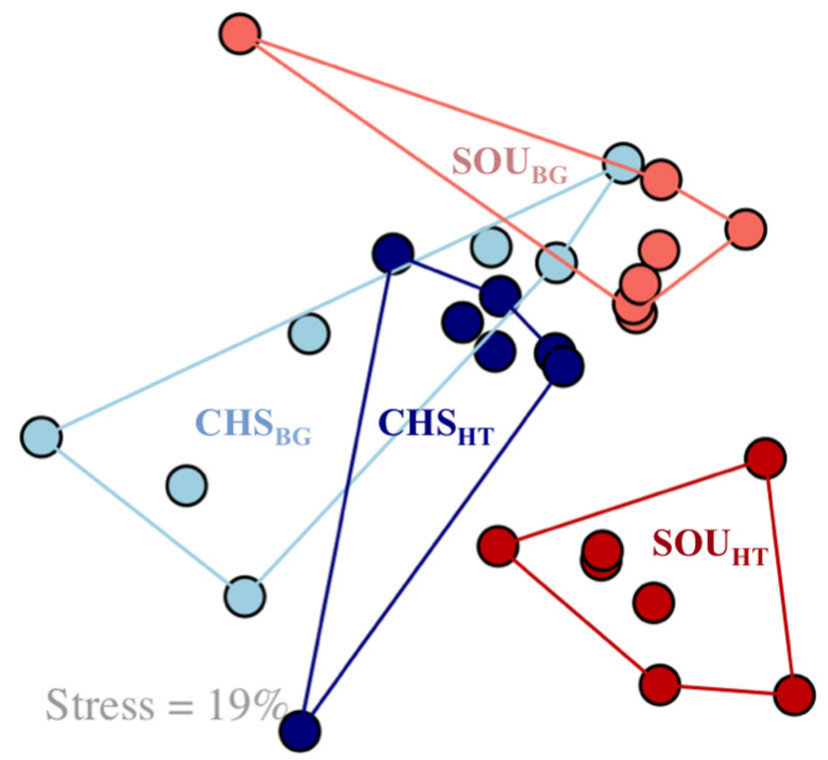
Nonmetric multidimensional scaling (NMDS) analysis, based on Bray-Curtis dissimilarity, revealing differences in the bacterial community structure between SOU_HT_ and all other investigated sites. Samples are color coded according to the sampling sites. Stress level = 19%.

The bacterial community structure of SOU_HT_ was statistically and significantly different compared to the near-by background site (SOU_BG_), as revealed by the NMDS analysis and the ANOSIM test (Figure 3, Table 3). In contrast, no such pattern was revealed at the Champagne area, where the bacterial communities of the hydrothermal and the background sites were highly similar and had overlapping structures. Furthermore, the structure of the bacterial communities at SOU_HT_ was statistically different compared to the other hydrothermal CHS_HT_ site.

**Table 3:** Analysis of Similarity (ANOSIM) test based on Bray-Curtis distance.

	**CHS_BG_**	**CHS_HT_**	**SOU_BG_**	**SOU_HT_**
CHS_BG_		0.152	0.137	0.008
CHS_HT_	0.3		0.004	0.004
SOU_BG_	0.3	0.6^*^		0.01
SOU_HT_	0.6^*^	0.6^*^	0.6^*^	

*ANOSIM R values are shown in the lower triangle and p-values in the upper triangle*.

* *Denotes significant separation of groups*.

**Figure 4:**
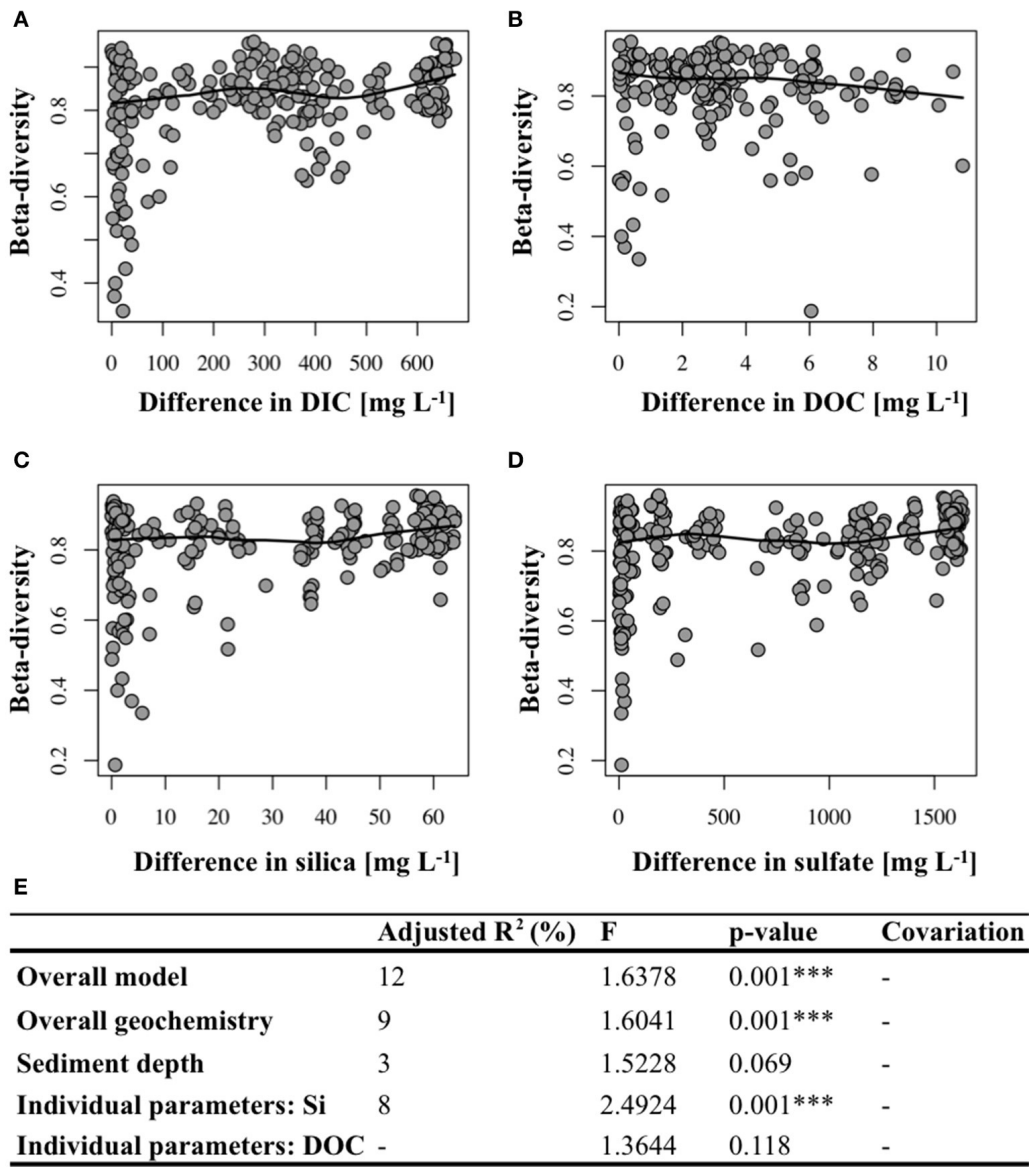
Correlation plots depicting positive relation between beta-diversity and differences in **(A)** DIC, **(C)** silica and **(D)** sulfate concentrations, but not **(B)** DOC (see also Supplementary Table 2 for associated Mantel test results). Solid line represents a LOWESS curve (locally weighted scatterplot smoothing). **(E)** Variation partitioning analysis. ^***^Denotes significant *p*-value.

Beta-diversity, i.e., differences in the microbial community structure between sites (including hydrothermal and background samples), was significantly correlated to differences in most of the investigated geochemical parameters, i.e., ${\text{SO}}_{4}^{2-}$, DIC, H_4_${\text{SiO}}_{4}^{◦}$, Cl^−^, K^+^, Mg^2+^ (Figures 4A–D, Supplementary Table 2) between the investigated sites. No significant correlation between beta-diversity and temperature could be found. The significant effect of the individual environmental factors on the bacterial community structure was confirmed by redundancy (RDA) and variation partitioning analysis (Figure 4E). The overall RDA model significantly explained 12% (*p*-value = 0.001) of the total variation in the bacterial communities at Dominica, of which 9% were due to overall differences in the sediment geochemistry between the sites (Figure 4E). Silica was the only important geochemical variable, which explained 8% (*p* = 0.001) of the variation in the bacterial community structures at Dominica. In contrast, DIC as well as the sediment depth did not significantly contribute to the overall model, and thus did not play any role in structuring the bacterial communities.

### Taxonomical composition of bacterial communities

Overall 58 phyla, 120 classes, and 732 genera were identified in this study. In general, slightly lower numbers of taxa were retrieved at the hydrothermal compared to their respective background sites at the taxonomical levels of class, family and genus (Supplementary Table 3).

**Figure 5:**
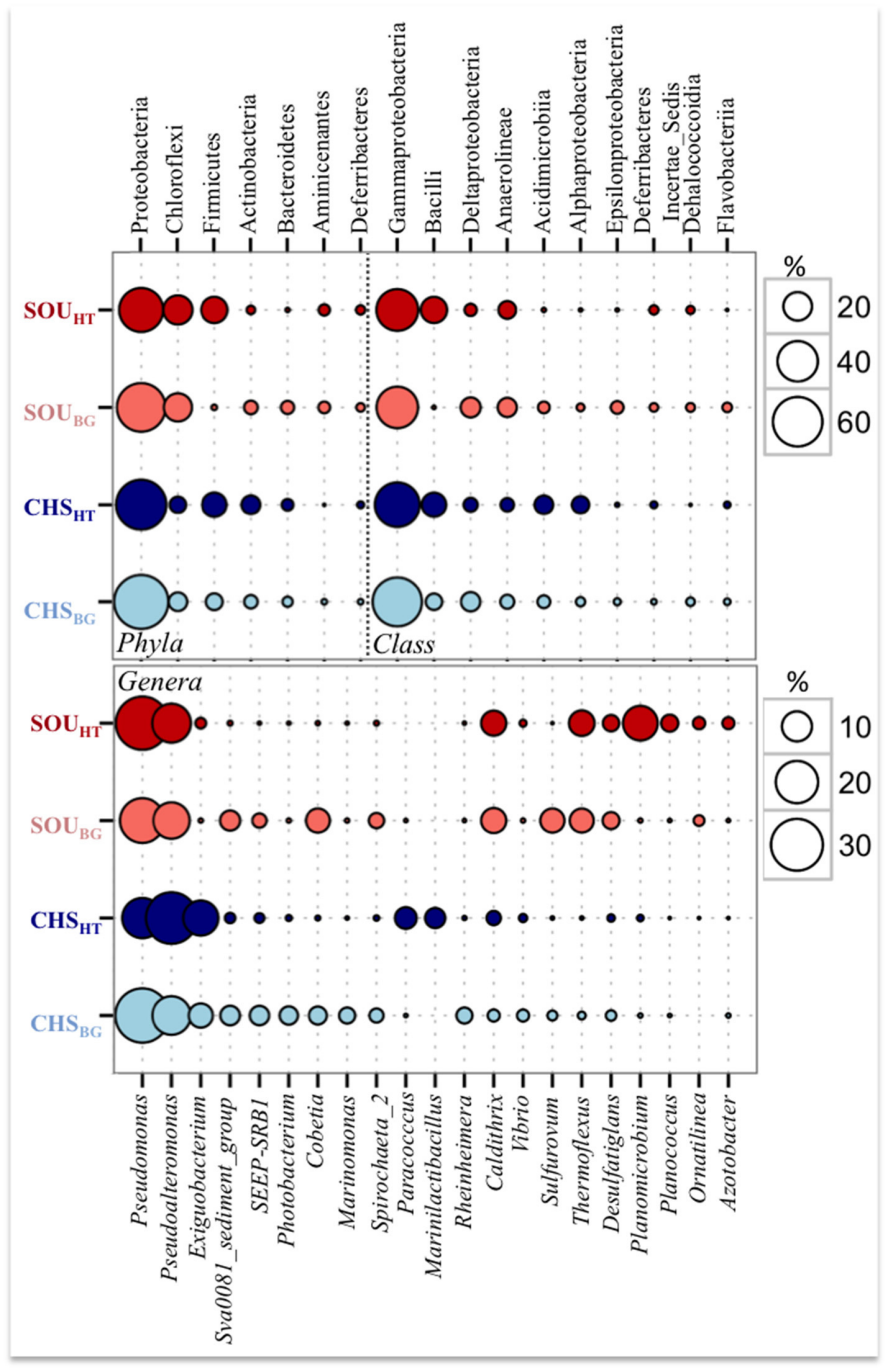
Comparison of the most sequence abundant phyla and classes (top five), as well as genera (top ten) at the hydrothermal and background sites.

All samples, including hydrothermal and background sites, were dominated by Proteobacteria (47–72%) and to lesser extent by Chloroflexi (7–21%) (Figure 5). Gammaproteobacteria was the most abundant class at all investigated sites in this study, comprising 59 % of the total number of sequences, (Figure 5). *Pseudomonas* (28%) and *Pseudoalteromonas* (26%) of Gammaproteobacteria were the most sequence abundant genera in the whole dataset. A large number of sequences belonging to these genera were related to *Pseudomonas zhaodongensis* (99.6% similarity) and *Pseudoalteromonas undina* (98.4% similarity; previously *Alteromonas undina*) (Table 4, Supplementary Table 4).

**Table 4:** Twenty most abundant sequences in the whole dataset, including information on their closest relatives and pairwise similarity.

**OTU ID**	**Sequence abundance**	**Relative sequence abundance (%)**	**Closest relative; accession number**	**Similarity (%)**	**Class**
CHS.BG.2.4.B_443194	18,916	0.9	*Exiguobacterium aurantiacum* (JNIQ01000001)	99.55	Bacilli
SOU.HV.8.10.B_5070135	16,274	0.8	*Pseudomonas zhaodongensis* (JQ762275)	99.55	Gammaproteobacteria
SOU.BG.0.2.B_4070160	14,290	0.7	*Pseudoalteromonas undina* (X82140)	95.98	Gammaproteobacteria
SOU.HV.0.2.B_3891936	13,250	0.6	*Planococcus maritimus* (CP016538)	98.43	Bacilli
CHS.HV.0.2.B_1132728	10,326	0.5	*Pseudoalteromonas arabiensis* (LRUF01000013)	99.11	Gammaproteobacteria
CHS.HV.16.18.B_7608234	10,130	0.5	*Pseudomonas songnenensis* (JQ762269)	98.88	Gammaproteobacteria
CHS.BG.2.4.B_5899776	9,769	0.5	*Exiguobacterium aurantiacum* (JNIQ01000001)	99.54	Bacilli
SOU.HV.0.2.B_5028604	9,294	0.4	*Planococcus maritimus* (CP016538)	99.77	Bacilli
CHS.HV.0.2.B_2873360	8,376	0.4	*Exiguobacterium aurantiacum* (JNIQ01000001)	99.54	Bacilli
CHS.HV.0.2.B_1963239	8,160	0.4	*Pseudomonas zhaodongensis* (JQ762275)	99.07	Gammaproteobacteria
SOU.HV.8.10.B_5611531	8,039	0.4	*Pseudomonas zhaodongensis* (JQ762275)	99.07	Gammaproteobacteria
SOU.BG.0.2.B_4110066	7,298	0.3	*Pseudoalteromonas undina* (X82140)	99.32	Gammaproteobacteria
SOU.BG.0.2.B_4517566	7,030	0.3	*Pseudoalteromonas nigrifaciens* (X82146)	100	Gammaproteobacteria
SOU.HV.8.10.B_3249484	6,952	0.3	*Pseudomonas zhaodongensis* (JQ762275)	99.78	Gammaproteobacteria
CHS.HV.16.18.B_5840744	6,669	0.3	*Pseudomonas songnenensis* (JQ762269)	99.31	Gammaproteobacteria
SOU.BG.0.2.B_5746454	6,277	0.3	*Pseudoalteromonas nigrifaciens* (X82146)	98.44	Gammaproteobacteria
SOU.HV.0.2.B_6185638	5,669	0.3	*Planococcus maritimus* (CP016538)	99.33	Bacilli
SOU.HV.0.2.B_4713558	5,667	0.3	*Planococcus maritimus* (CP016538)	99.76	Bacilli
CHS.BG.2.4.B_6818917	5,468	0.3	*Pseudoalteromonas undina* (X82140)	99.78	Gammaproteobacteria
SOU.HV.8.10.B_3312652	5,390	0.3	*Pseudomonas zhaodongensis* (JQ762275)	99.54	Gammaproteobacteria

Differences between the hydrothermal and the background sites were larger at the Soufrière compared to Champagne at all taxonomical levels (Figure 5). SOU_HT_ had substantially higher abundance of Bacilli and Deferribacteres, and low proportion of Actinobacteria, Bacteroidetes, and Epsilonproteobacteria compared to all other sites. In contrast, Deltaproteobacteria, Acidimicrobiia, and Alphaproteobacteria classes had relatively higher abundance in background sediments, but also at the Champagne hydrothermal site.

Few of the most abundant sequences belonged to Bacilli, and were closely related to *Planococcus maritimus* (99.5% similarity), *Exiguobacterium aurantiacum* (99.7% similarity) and *Marinilactibacillus piezotolerans* (98.7% similarity) (Supplementary Table 4). Sequences related to *Planococcus maritimus* were substantially more abundant at SOU_HT_, while those related to *Exiguobacterium aurantiacum* and *Marinilactibacillus piezotolerans* dominated the CHS_HT_. Other abundant genera at SOU_HT_ included *Thermoflexus* (Chloroflexi), *Caldithrix* (Calditrichaeota), *Desulfatiglans* and *Dissulfurirhabdus* (Deltaproteobacteria).

Differences in the taxonomical composition between the two hydrothermal sites were larger compared to the differences between the background sites, as only 26% of the total genera were found at both hydrothermal sites, while more than double (61%) were shared by the two background sites. Of the most sequence abundant genera, *Caldithrix* and *Exiguobacterium*, along with *Pseudomonas* and *Pseudoalteromonas*, were found at both hydrothermal sites but with substantially different sequence abundances. Highly abundant genera at the background sediments included two genera of the Desulfobacteraceae (Sva0081_sediment group and SEEP-SRB1), *Cobetia* (Gammaproteobacteria), *Spirochaeta* and *Sulfurovum* (Epsilonproteobacteria).

## Discussion

### Shallow-water hydrothermal systems as islands of distinct physicochemical conditions within the coastal ocean

In this study we investigated seawater, hydrothermal fluid and sediment porewater at two sites with hydrothermal activity, the Champagne Hot Springs (CHS_HT_) and Soufrière Bay (SOU_HT_). The two sites were located at approximately 5 m water depth, and compared to other sites of hydrothermal activity, had intermediate temperatures between 46°C and 75°C (e.g., Pichler and Veizer, 1999). Their pH was slightly acidic and their chemical composition was intermediate between their respective end-members (Supplementary Figure 3). This position indicates that the hydrothermal fluids are a mixture of meteoric water and seawater (e.g., McCarthy et al., 2005; Gomez-Saez et al., 2015). As shown for other shallow-water hydrothermal systems (e.g., Wenzhöfer et al., 2000; Price et al., 2007; Yücel et al., 2013), mixing with hydrothermal fluids substantially altered the porewater geochemistry, which created distinct island-like habitats characterized by elevated temperatures (42°C to 56°C), higher amounts of H_4_${\text{SiO}}_{4}^{◦}$ and H_3_${\text{BO}}_{4}^{◦}$ and lower concentrations of ${\text{SO}}_{4}^{2-}$, Cl^−^, K^+^, Mg^2+^, and Na^+^ in comparison to the surrounding sediments (Figure 2, Supplementary Figure 2). The porewater was also enriched with electron donors, i.e., Fe^T^ and to a certain extent totS^2−^ (this study and McCarthy et al., 2005), as well as DIC, which can in turn promote microbial processes such as chemotrophy, as well as overall benthic primary productivity. In line with this, Kleint et al. (2017) found that the fluids of Dominica were highly enriched in stabilized and bioavailable Fe^2+^, due to same mechanisms detected at deep-sea hydrothermal systems (Bennett et al., 2008; Hawkes et al., 2013), i.e., strong complexation of Fe^2+^ with dissolved organic carbon. Upon contact with oxygenated seawater, iron precipitated out of the fluids, and formed extensive hydrous ferric oxide reddish layers that covered the venting areas (Figure 1), similar to the ones found at deep-sea (Fleming et al., 2013) and other shallow-water hydrothermal systems (e.g., Pichler and Veizer, 1999; Handley et al., 2010). These hydrothermal precipitates represent energy-rich solid surfaces that provide micro-niches and energy sources to microorganisms, otherwise not present at the background sediments of Dominica or, generally, in coastal sediments.

Differences in the overall porewater geochemistry between the two hydrothermal sites were higher than between the two background sites (Supplementary Figure 1), indicating that activity of shallow-water hydrothermal systems can contribute substantially to the physicochemical and biogeochemical heterogeneity of coastal sediments.

End-member analysis revealed that fluids at both hydrothermal sites were the product of mixing between meteorically derived hydrothermal fluids (i.e., Sulfur Springs) and seawater, and that the amount of mixing between hydrothermal fluid and seawater controlled the geochemistry of the porewater (see also McCarthy et al., 2005; Supplementary Figure 3). Despite the geographic proximity and the same origin of the hydrothermal fluids, Champagne and Soufrière hydrothermal systems had substantially different porewater geochemistry (Figure 2, Supplementary Figure 1). Silica and Mg^2+^ concentrations are generally excellent indicators for the amount of hydrothermal component in a mixture between seawater and hydrothermal fluid (e.g., Pichler and Veizer, 1999). The hydrothermal fluid at SOU_HT_ had a higher temperature, higher H_4_${\text{SiO}}_{4}^{◦}$ and lower Mg^2+^ concentrations, which indicated a larger hydrothermal component at this system compared to CHS_HT_. This observation was also true for the hydrothermally-influenced porewater at both sites. The hydrothermal component in the sediment porewater was larger at SOU_HT_ than at CHS_HT_. These differences are likely due to greater hydrothermal activity (as evidenced by more gas bubbling) or decreased rates of mixing of fluids with seawater due to finer sediments at SOU_HT_ (Supplementary Figure 4). The pronounced difference in Ca^2+^ between the two sites, i.e., CHS_HT_ had higher concentrations than can be explained by the mixing model (Supplementary Figure 3A), is likely a result of the proximity of CHS_HT_ to a coral reef and thus abundance of CaCO_3_ minerals in the sediment. At a pH of 6.3 aragonite and calcite are readily dissolved (Pichler and Veizer, 2004). The same behavior could also be seen for the variation of Sr^2+^, which is also supplied to the porewater and hydrothermal fluid through the dissolution of CaCO_3_ minerals (Supplemental Figure 3A). The deviation of ${\text{SO}}_{4}^{2-}$ values for Sulfur Springs from the respective CHS_HT_ and SOU_HT_ mixing lines (Supplemental Figure 3) is caused by oxidation of H_2_S and SO_2_ to ${\text{SO}}_{4}^{2-}$ by atmospheric oxygen. Sulfur Springs discharges inland and thus its hydrothermal fluid has extensive contact with the atmosphere prior to sampling. At the SOU_HT_ site precipitation of gypsum (CaSO_4_) and thus removal of ${\text{SO}}_{4}^{2-}$ could have an additional effect on the ${\text{SO}}_{4}^{2-}$ concentration. This, however, was not directly observed, due to the lack of a detailed mineralogical examination. Nevertheless, it is common occurrence because, above 70°C fluid temperature gypsum starts to precipitate in shallow-water hydrothermal systems (Pichler and Humphrey, 2001).

These results are in line with previous studies, which also showed large physicochemical and biogeochemical variations among individual hydrothermal sites (e.g., Zhang et al., 2012; Price et al., 2013b; Lentini et al., 2014). Overall, these findings reveal large variability, not only among shallow-water hydrothermal systems of different regions, but also at small spatial scales within a single volcanic region.

Most of the geochemical parameters measured at the two Dominica hydrothermal sites fall within the lower range compared to values reported from other shallow-water hydrothermal systems, i.e., off Panarea Island (Italy) (Maugeri et al., 2010), Milos Island (Greece) (Wenzhöfer et al., 2000; Price et al., 2013a; Yücel et al., 2013) and Kueishan Island (Taiwan) (Zhang et al., 2012; Wang et al., 2015). In line, although SOU_HT_ is regarded as iron-rich (Gomez-Saez et al., 2017), the measured iron concentrations were a magnitude lower compared to similar sites elsewhere (Handley et al., 2010; Hoshino et al., 2016). Despite this, the measured DIC concentrations at CHS_HT_ and SOU_HT_ were up to five times higher than in other shallow-water hydrothermal systems (e.g., Robinson et al., 1997; Wenzhöfer et al., 2000; Zhang et al., 2012). These settings can thus potentially provide conditions for a comparatively enhanced photo- and chemolithotrophic production of organic matter, and consequently base for heterotrophic processes in the sediments of the Dominica shallow-water hydrothermal system.

### Bacterial communities of different habitats at dominica

Hydrothermal systems are regarded as extreme chemosynthetic environments because of the high levels of toxic metals in the emerging fluids, elevated temperatures, increased sulfide concentrations and often low pH (Amend and Shock, 1998; Tarasov et al., 2005; Price et al., 2013b). These extreme environmental conditions pose stress to many organisms and are believed to depress their ability to function (Rothschild and Mancinelli, 2001), resulting in a reduced number of highly adapted and specialized organisms which are able to inhabit these extreme environments. Thus, low diversity and high biomass of deep-sea hydrothermal systems compared to their surroundings is thought to be a universal phenomenon, applicable to all organismal groups from meio- and macro- to megafauna, found at extreme environments (Grassle and Maciolek, 1992; Van Dover, 2000; Ramirez-Llodra et al., 2007).

Here, we show that microorganisms follow the same pattern, in that both Champagne and Soufrière hydrothermal systems had significantly lower alpha diversity, as well as a lower number of taxa for most of the investigated taxonomical levels compared to the respective background sediments (Table 2). This is in agreement with previous studies, which has revealed lower diversity at the shallow-water vent outlet compared to the transition zones in the Aegean Sea (Sievert et al., 2000; Giovannelli et al., 2013). A contrasting trend was shown for the Espalamaca shallow-water hydrothermal system (Rajasabapathy et al., 2014), however this study used cultivation techniques, which are known to bias the diversity of microbial communities.

At both hydrothermal sites of Dominica, alpha diversity did not change with sediment depth, while at the background sediments a tentative increase with sediment depth was observed (Table 2). This result indicates that, unlike at coastal sediments (Böer et al., 2009 and references therein), at extreme chemosynthetic environments in general, i.e., at hydrothermal systems (this study) and cold seeps (Pop Ristova et al., 2012, 2015) the sediment depth likely does not influence the richness of the microbial communities. This is likely due to the flushing of sediments with fluids and the highly variable geochemistry of these ecosystems.

Only the Soufrière hydrothermal site, which was strongly influenced by hydrothermal fluids, bore distinct communities compared to the background sediments (Figure 3). At the less hydrothermally-impacted Champagne site, the community was more similar to the background sediments, likely due to the small differences in geochemistry between these two habitats. An alternative explanation for such high overlap in the bacterial community structure between the different habitats at Champagne can be the fact that the background site was located within a larger area of hydrothermally-impacted sediments, thus potentially representing a past or a temporally inactive hydrothermal site.

Despite differences in geochemistry and temperatures (29°C at the background and 56°C at the hydrothermal site at 25 cm), all hydrothermal and background samples were dominated by Gammaproteobacteria sequences related to *Pseudomonas zhaodongensis* and *Pseudoalteromonas undina* (Figure 5, Table 4). Similarly, *Pseudoalteromonas undina* has been has been previously isolated from a hydrothermal vent in the deep-sea (Rougeaux et al., 1996). *Pseudomonas zhaodongensis* is known from saline and alkaline soils (Zhang et al., 2015c), and has not been so far reported from shallow-water hydrothermal systems. However, several other species of the *Pseudomonas* genus have been isolated from deep-sea hydrothermal vents and hot springs (Wang et al., 2002; Liu et al., 2013; Peña et al., 2013). Due to their high genetic and metabolic potential for adaptation to different environmental conditions, e.g., variable temperature and pH regimes, these two genera can inhabit many diverse habitats ranging from soil to marine ecosystems, including also extreme environments such as hydrothermal vents (Mihailov et al., 2006; Mohandass et al., 2012). This can explain their high abundances in both hydrothermal and background sediments of Dominica.

Variation in the abundances of bacterial taxa between habitats, rather than the sheer presence or absences of taxonomical types, was found to be mainly responsible for the observed differences between hydrothermal and background sites (Figure 5). In comparison to background sediments, hydrothermal sites had relatively lower abundance of Deltaproteobacteria, Acidimicrobiia, and Alphaproteobacteria, but higher numbers of Bacilli and Deferribacteres. The latter two classes are common members of bacterial communities of deep and shallow-water hydrothermal systems, and similarly to this study have been found in larger numbers directly at the vents or at sites with higher temperatures (Takai et al., 2003; Vetriani et al., 2005; Handley et al., 2010; Takaki et al., 2010; Gugliandolo et al., 2012; Mohandass et al., 2012; Lentini et al., 2014; Rajasabapathy et al., 2014; Zhang et al., 2015b). Overall, the majority of bacteria detected in this study could inhabit wide ranges of temperatures, likely as an adaptation to the temporarily and spatially variable geochemical conditions at the Dominica hydrothermal systems.

### Bacterial communities of individual shallow-water hydrothermal systems at dominica

Epsilonproteobacteria and sulfur-oxidizers of Gammaproteobacteria have been identified as the most widespread and abundant bacterial groups at shallow-water hydrothermal systems worldwide (Price and Giovannelli, 2017 and references therein). This is in striking contrast to our findings at the Dominica shallow-water systems, where Epsilonproteobacteria as well as other chemolithotrophs were only present in low numbers, and thus likely played a relatively smaller role at this ecosystem (Figure 5). In contrast to all other known shallow-water hydrothermal systems, Dominica hydrothermal sites were dominated by heterotrophic Gammaproteobacteria.

Despite differences in the sediment geochemistry, both investigated Dominica hydrothermal systems showed similar levels of estimated species richness (Table 2), with only slightly lower alpha diversity values detected at the cooler Champagne hydrothermal site (Shannon diversity CHS_HT_ = 7 ± 1, SOU_HT_ = 7.3 ± 0.5). Comparable estimated species richness have been reported from other shallow-water hydrothermal systems in the Mediterranean and off Taiwan (Shannon diversity 6–9.2) (Lentini et al., 2014; Wang et al., 2015). Based on Chao1 index for estimated species richness, Dominica shallow-water systems harbored higher alpha-diversity compared to their deep-sea counterparts (Ruff et al., 2015).

Reflecting on the heterogeneity in the porewater geochemistry, Champagne and Soufrière hydrothermal sites harbored bacterial communities with significantly different structures (Figure 3). Differences in the bacterial community structure were significantly correlated to variations in the porewater geochemistry and the magnitude of hydrothermal fluid influence (Figure 4). This expands the findings of previous studies that could show an indirect link between variations in the microbial communities and changes in sediment temperature at shallow-water hydrothermal systems (Sievert et al., 1999; Giovannelli et al., 2013). More specifically, the results of this study indicate that likely not only the rate of fluid flow, but more specifically the rate of mixing of hydrothermal fluids with seawater, which in itself depends on the grain size of the sediment (Supplementary Figure 4), can profoundly influence the structure of shallow-water hydrothermal microbial communities. Sediments at the SOU_HT_ were more compact due to the smaller grain size, which impedes the advection of fluids and protrusion of seawater (Bear, 2013). Porewater geochemical parameters measured in this study explained overall a relatively small portion of the total variation in the bacterial community structure (12%; Figure 4E), indicating that other, yet unidentified factors, have an important influence on the microbial community structure of the shallow-water hydrothermal systems of Dominica.

Many of the bacterial taxa detected here were found over the entire range of measured temperatures (38–56°C), but had substantially different relative abundances at the individual hydrothermal sites and thus likely preferred niches with different temperature and specific geochemical conditions (Figure 5). Actinobacteria (Acidimicrobiia) and Alphaproteobacteria, which have been found at hydrothermal systems with higher temperatures (Lentini et al., 2014; Qin et al., 2015), here dominated the cooler Champagne site and favored sediments with relatively lower impact by hydrothermal fluids. In contrast, Chloroflexi (*Anaerolinea* and *Thermoflexia*), Aminicenantes, *Nitrospira, Caldithrix*, and *Desulfatiglans* were more numerous at the Soufrière site characterized by higher temperatures (47–56°C) and larger hydrothermal impact. These taxa are known from hot springs, deep and shallow-water hydrothermal systems with variable temperatures (Miroshnichenko et al., 2003, 2010; Davis and Moyer, 2008; Lebedeva et al., 2011; Nunoura et al., 2013; Farag et al., 2014; Lentini et al., 2014; Teske et al., 2014; Thiel et al., 2016; Cerqueira et al., 2017). Similar to other shallow-water hydrothermal systems, Bacilli were highly numerous and comprised the second most abundant class at both hydrothermal sites of Dominica (Dando et al., 1998; Maugeri et al., 2010; Cihan et al., 2012; Gugliandolo et al., 2012; Price et al., 2013a; Lentini et al., 2014). They were represented by sequences mainly related to *Exiguobacterium aurantiacum, Marinilactibacillus piezotolerans*, and *Planococcus maritimus*. This presents a first report of their occurrence in hydrothermal environments, however, their genera i.e., *Exiguobacterium* and *Planococcus* encompass numerous meso- and thermophilic members that have been identified or isolated from shallow-water and deep-sea hydrothermal vents (Crapart et al., 2007; Vishnivetskaya et al., 2009; Maugeri et al., 2010; Zhang et al., 2015a; Chen and Sun, 2017). At Dominica, different hydrothermal sites were dominated by different Bacilli genera: sequences related to *Exiguobacterium* and *Marinilactibacillus* dominated the cooler Champagne site, while *Planococcus* was among the 10 most abundant genera at the hotter Soufriére site. This result suggests an ecological species replacement of microbial members of the Bacilli at shallow-water hydrothermal systems, over a range of 38–56°C temperatures.

With only 26% of the genera common to both hydrothermal sites, while more than 61% to both background sites, each individual shallow-water hydrothermal site of Dominica hosted more unique diversity compared to background sediments. This study demonstrates that each individual shallow-water hydrothermal site within the larger hydrothermal area contributes to the overall biodiversity of coastal sediments.

### Metabolic potential of the bacterial communities at dominica shallow-water hydrothermal systems

Existence of steep geochemical and physical gradients, availability of different substrates in the hydrothermal fluids, as well as light and oxygen provide a variety of microniches to metabolically diverse microorganisms at shallow-water hydrothermal systems (e.g., Giovannelli et al., 2013). At the Dominica shallow-water systems we found high taxonomical diversity fueled by a heterogeneous underlying geochemistry, suggesting a versatile metabolic potential of the microbial communities at these sites.

Sulfide-oxidizing Epsilonproteobacteria represent the most dominant and ubiquitous group at shallow-water and deep-sea hydrothermal systems (Teske and Reysenbach, 2015 and references therein). At Dominica hydrothermal systems, we found Epsilonproteobacteria related to sequences from a sulphidic cold-seep site in Japan (95.9% similarity) as well as the sulfide-oxidizing *Sulfurimonas autotrophica* (98.9% similarity) (Inagaki et al., 2003; Arakawa et al., 2005) (Supplementary Table 4). However, in Dominica hydrothermal sediments, Epsilonproteobacteria comprised overall only a minor component of the hydrothermal communities (<1%). Although some sulfide was present in the venting fluids of Dominica (0.44 mg L^−1^; Gomez-Saez et al., 2015), likely it was not entirely available to the microbial communities. Sulfide could have escaped as a gas from the system (McCarthy et al., 2005) or it could have precipitate with Fe^2+^ to form iron sulfide and pyrite, which could in turn explain the low abundances of chemolithotrophic sulfide-oxidizers detected in this study.

Other chemolithotrophs present at the Dominica hydrothermal sites included microorganisms distantly related to *Mariprofundus ferrooxydans* (95.1% similarity; Zetaproteobacteria), which use reduced iron as their source of energy (Emerson et al., 2007, 2010) (Supplementary Table 4). The surface sediment layers of Dominica hydrothermal sites, where both Fe^2+^ from the fluids and oxygen from the seawater are available (see Figure 2), provided suitable environments for these microorganisms. *Mariprofundus ferrooxydans* has a global distribution and it has been reported from various habitats, ranging from deep-sea and shallow-water hydrothermal systems to coastal and brackish sediments (Handley et al., 2010; McAllister et al., 2011; McBeth et al., 2011; Ionescu et al., 2015; Hoshino et al., 2016). The number of sequences related to *Mariprofundus ferooxydans* detected here were rather low (<1%), however they showed increased abundances and were among the 10 most abundant genera in experiments using sediments from the same sites at Dominica and incubated with ^13^C-bicarbonate (Gomez-Saez et al., 2017). The same study detected even higher diversity of bacteria potentially involved in the cycling of iron, suggesting that microbial iron cycling plays an important role in the biogeochemistry of the Dominica shallow-water hydrothermal system. In line with this, at the level of the whole bacterial community, which was dominated by Chloroflexi *(Anaerolinea)*, Deltaproteobacteria, Actinobacteria, Aminicenantes, and *Caldithrix*, the Dominica hydrothermal systems mostly resembled an iron-rich shallow-water hydrothermal sites off Japan (Hoshino et al., 2016) and to a certain extent off Santorini (Handley et al., 2010). Further chemolithotrophic taxa detected in this study belonged to *Dissulfurirhabdus*, which represented one of the 10 most abundant genera at the hydrothermal sites of Dominica. Sequences of this genus were distantly related to *Dissulfurirhabdus thermomarina* (88.5% similarity), a thermophilic microorganism that grows chemolithotrophically using molecular hydrogen and has been previously isolated from a shallow-water hydrothermal vent off Kuril Island in Russia (Slobodkina et al., 2016).

Although fluids of hydrothermal ecosystems are often depleted in sulfate (Tivey, 2007), due to mixing with seawater, the surface sediments of Dominica were not limited by sulfate and contained sufficient concentrations (Figure 2) to support microorganisms with sulfur-based metabolisms. Concomitantly, here we identified sequences distantly affiliated to sulfate-reducers *Desulfatiglans aniline* (91% similarity; Suzuki et al., 2014) and SEEP-SRB1 (98% similarity) of the Deltaproteobacteria (Supplementary Table 4). They were among the ten most abundant genera at the hydrothermal sites, but had highly variable abundances in the different hydrothermal samples (<1–10%; Figure 5). *Desulfatiglans aniline* and SEEP-SRB1 are widespread sulfate reducers that inhabit various chemosynthetic ecosystems, including hydrothermal vents, but also coastal and pelagic sediments, where they are involved in parts of the cycling of sulfur (Handley et al., 2010; Yanagawa et al., 2012; Frank et al., 2013; Pop Ristova et al., 2015, 2017a; Hoshino et al., 2016).

In comparison to other known shallow-water hydrothermal sites, a unique feature of the Dominica hydrothermal sites was the clear dominance of Gammaproteobacteria (encompassing 59 % of the sequences in the whole dataset), represented mainly by the two heterotrophic genera related to *Pseudoalteromonas undina* (26%) and *Pseudomonas zhaodongensis* (28%). In laboratory conditions both *Pseudoalteromonas undina* and *Pseudomonas zhaodongensis* display chemo-organotrophic characteristics (Chan et al., 1978; Zhang et al., 2015c). This is in line with field studies, which have shown that these genera occupy heterotrophic niches at hydrothermal ecosystems and are responsible for the reduction of various metals (Wang et al., 2002; Mihailov et al., 2006; Rathgeber et al., 2006; Peña et al., 2013). In addition, we have detected high abundance of heterotrophic *Anaerolinea* mainly related to sequences from Fe-rich hydrothermal sediments (Sekiguchi et al., 2003; Forget et al., 2010; Nunoura et al., 2013; Podosokorskaya et al., 2013), as well as sequences related to the heterotrophic *Paracoccus sediminis* (Pan et al., 2014) and the thermophilic chemo-organotrophic bacterium *Caldithrix paleochoryensis* isolated from a shallow-water hydrothermal site in Milos, Greece (97.3% similarity; Miroshnichenko et al., 2010) (Supplementary Table 4). Overall, these heterotrophic taxa, including *Exiguobacterium, Planococcus*, and *Marinilactibacillus* bacilli (Wei et al., 2017), comprised 78% of the sequences in the whole dataset. Clear predominance of heterotrophs indicates that heterotrophy is likely the most dominant metabolism at the hydrothermal systems of Dominica. This may be explained by the fact that both sites were anthropogenically influenced and were located in the vicinity of the coast (5–10 m away), as well as near a river outlet in the case of the Soufrière site. Such conditions are known to enhance heterotrophic processes at shallow-water hydrothermal systems, due to the extra allochthonous input of organic matter (Sievert et al., 2000). Therefore, Dominica shallow-water hydrothermal systems represent an ideal natural laboratory for the study of heterotrophy and its associated microorganisms under high temperatures, which remain understudied compared to autotrophic processes and specialist thermophilic bacteria (Mohandass et al., 2012).

Although heterotrophic microorganisms represented the largest and most important fraction of the microbial communities in this study, we could not detect any correlation between the concentration of dissolved organic carbon and the overall structure of microbial communities at the Dominica hydrothermal sites (Figure 4). Potentially, other aspects of the complex dissolved organic matter, e.g., the quality, which gets altered during hydrothermal circulation (Hawkes et al., 2014), and/or the composition or concentration of the individual organic components, might be more relevant factors for the structuring of the microbial communities. These should be the focus of future studies aiming to better understand heterotrophic processes and its corresponding microorganisms at hydrothermal ecosystems.

## Author contributions

PP and SIB designed the research. PP, SIB, and TP carried out field sampling. PP and TP performed laboratory work. PP analyzed data and wrote the manuscript with help and input from SIB, TP, and MWF.

### Conflict of interest statement

The authors declare that the research was conducted in the absence of any commercial or financial relationships that could be construed as a potential conflict of interest.
