# Supplementary material for: Bacterial Diversity and Biogeochemistry of Two Marine Shallow-Water Hydrothermal Systems off Dominica (Lesser Antilles)
Source: Front Microbiol. 2017 Dec 4;8:2400. doi: 10.3389/fmicb.2017.02400 (PMC5722836; doi:10.3389/fmicb.2017.02400)
Supplement: Supplementary file 1 [file Table1.PDF]

SUPPLEMENTARY TABLE 1. Correlation analyses (Pearson coefficient) between individual geochemical parameters. r-correlation coefficient is shown in the lower triangle and p-values in the upper triangle. Bolded values represent significant correlations.

	Cl^-	SO_4^{2-}	Fe^{2+}	K^+	Mg^{2+}	Si	DOC	Temperature
Cl^-		**0.000**	**0.000**	**0.000**	**0.000**	**0.000**	0.326	**0.000**
SO_4^{2-}	**1.0**		**0.000**	**0.000**	**0.000**	**0.000**	0.298	**0.000**
Fe^{2+}	**-0.8**	**-0.8**		**0.000**	**0.000**	**0.000**	0.616	**0.032**
K^+	**1.0**	**1.0**	**-0.8**		**0.000**	**0.000**	0.510	**0.000**
Mg^{2+}	**1.0**	**1.0**	**-0.8**	**1.0**		**0.000**	0.582	**0.000**
**Si**	**-1.0**	**-1.0**	**0.8**	**-1.0**	**-1.0**		0.390	**0.000**
**DOC**	-0.2	-0.2	0.1	-0.1	-0.1	0.2		0.418
**Temperature**	**-1.0**	**-1.0**	**0.6**	**-1.0**	**-1.0**	**0.9**	0.3	
