# Supplementary material for: Bacterial Diversity and Biogeochemistry of Two Marine Shallow-Water Hydrothermal Systems off Dominica (Lesser Antilles)
Source: Front Microbiol. 2017 Dec 4;8:2400. doi: 10.3389/fmicb.2017.02400 (PMC5722836; doi:10.3389/fmicb.2017.02400)
Supplement: Supplementary file 2 [file Table2.PDF]

SUPPLEMENTARY TABLE 2. Mantel test based on spearman coefficient. Testing for correlation between beta-diversity (calculated as Bray-Curtis dissimilarities) and difference in individual geochemical parameters. Bolded values denote significant correlation.

	**Spearman's coefficient r**	**p-value**
**Overall geochemistry**	**0.3**	**0.003**
**Chloride**	**0.3**	**0.005**
**Sulphate**	**0.3**	**0.005**
**Iron**	0.1	0.107
**Potassium**	**0.3**	**0.002**
**Magnesium**	**0.3**	**0.003**
**Silica**	**0.2**	**0.018**
**Dissolved Organic Carbon**	-0.07	0.711
**Dissolved Inorganic Carbon**	**0.2**	**0.003**
**Temperature**	0.1	0.216
