# Supplementary material for: Bacterial Diversity and Biogeochemistry of Two Marine Shallow-Water Hydrothermal Systems off Dominica (Lesser Antilles)
Source: Front Microbiol. 2017 Dec 4;8:2400. doi: 10.3389/fmicb.2017.02400 (PMC5722836; doi:10.3389/fmicb.2017.02400)
Supplement: Supplementary file 3 [file Table3.PDF]

SUPPLEMENTARY TABLE 3. Number of taxonomical levels identified at all investigated sites.

	Number of phyla	Number of classes	Number of families	Number of genera
**CHS_{HT}**	53	101	268	512
**CHS_{BG}**	53	107	270	516
**SOU_{HT}**	53	104	247	463
**SOU_{BG}**	54	109	282	553
