# Supplementary material for: Bacterial Diversity and Biogeochemistry of Two Marine Shallow-Water Hydrothermal Systems off Dominica (Lesser Antilles)
Source: Front Microbiol. 2017 Dec 4;8:2400. doi: 10.3389/fmicb.2017.02400 (PMC5722836; doi:10.3389/fmicb.2017.02400)
Supplement: Supplementary file 4 [file Table4.PDF]

**SUPPLEMENTARY TABLE 4.** Excerpt of sequences belonging to the most abundant and other genera, and their top blast hit (closest relative and closest uncultured relative).

| OTU ID | Class | Sequence abundance | Relative sequence abundance ( %) | Closest relative; Accession Number | Similarity (%) | Closest cultured relative; Accession Number | Similarity (%) |
|---|---|---|---|---|---|---|---|
| CHS.BG.2.4.B_1002998 | Anaerolineae | 718 | 0.03 | Uncultured Anaerolinaeles (FJ905697) | 95.52 | Ornatilinea apprima (LGCL01000043) | 87.26 |
| CHS.BG.16.18.B_3049345 | Anaerolineae | 614 | 0.03 | Uncultured Anaerolinaeles (DQ811856) | 98.82 | Ornatilinea apprima (LGCL01000043) | 88.68 |
| CHS.HV.2.4.B_731535 | Anaerolineae | 318 | 0.02 | Uncultured Anaerolinaeles (EU266884) | 94.34 | Ornatilinea apprima (LGCL01000043) | 89.15 |
| SOU.HV.8.10.B_138425 | Calditrichae | 1078 | 0.05 | Caldithrix palaeochoryensis (FJ999729) | 98.14 | Caldithrix palaeochoryensis (FJ999729) | 98.14 |
| SOU.HV.16.18.B_7265846 | Calditrichae | 378 | 0.02 | Caldithrix palaeochoryensis (FJ999729) | 96.53 | Caldithrix palaeochoryensis (FJ999729) | 96.53 |
| SOU.HV.8.10.B_5541144 | Calditrichae | 243 | 0.01 | Caldithrix palaeochoryensis (FJ999729) | 97.37 | Caldithrix palaeochoryensis (FJ999729) | 97.37 |
| SOU.HV.8.10.B_5077231 | Deltaproteobacteria | 119 | 0.01 | Uncultured Desulfatiglans (JN514781) | 92.86 | Desulfatiglans anilini (AULM01000006) | 89.96 |
| PM.2.4.B_5774565 | Deltaproteobacteria | 182 | 0.01 | Uncultured Desulfatiglans (AB722136) | 93.53 | Desulfatiglans anilini (AULM01000006) | 90.85 |
| SOU.HV.16.18.B_2524122 | Deltaproteobacteria | 96 | 0.00 | Uncultured Desulfatiglans (JN514781) | 96.65 | Desulfatiglans anilini (AULM01000006) | 92.86 |
| CHS.HV.16.18.B_7268371 | Epsilonproteobacteria | 829 | 0.04 | Uncultured Arcobacter (AB189374) | 95.98 | Arcobacter bivalviorum (FJ573217) | 93.14 |
| PM.2.4.B_7018731 | Epsilonproteobacteria | 288 | 0.01 | Sulfurimonas autotrophica (CP002205) | 99.05 | Sulfurimonas autotrophica (CP002205) | 99.05 |
| SOU.BG.0.2.B_3306566 | Epsilonproteobacteria | 186 | 0.01 | Sulfurimonas autotrophica (CP002205) | 98.82 | Sulfurimonas autotrophica (CP002205) | 98.82 |
| CHS.HV.0.2.B_2873360 | Bacilli | 8376 | 0.40 | Exiguobacterium aurantiacum (JNIQ01000001) | 99.54 | Exiguobacterium aurantiacum (JNIQ01000001) | 99.54 |
| CHS.HV.0.2.B_1963239 | Bacilli | 8160 | 0.39 | Exiguobacterium aurantiacum (JNIQ01000001) | 99.11 | Exiguobacterium aurantiacum (JNIQ01000001) | 99.11 |
| CHS.HV.0.2.B_1513738 | Bacilli | 5084 | 0.24 | Exiguobacterium aurantiacum (JNIQ01000001) | 99.78 | Exiguobacterium aurantiacum (JNIQ01000001) | 99.78 |
| CHS.HV.2.4.B_6115545 | Bacilli | 1040 | 0.05 | Marinilactibacillus piezotolerans (jgi.1067996) | 99.33 | Marinilactibacillus piezotolerans (jgi.1067996) | 99.33 |
| CHS.HV.2.4.B_1311455 | Bacilli | 452 | 0.02 | Marinilactibacillus piezotolerans (jgi.1067996) | 98.44 | Marinilactibacillus piezotolerans (jgi.1067996) | 98.44 |
| CHS.HV.2.4.B_7098925 | Bacilli | 321 | 0.02 | Marinilactibacillus piezotolerans (jgi.1067996) | 97.71 | Marinilactibacillus piezotolerans (jgi.1067996) | 97.71 |
| CHS.HV.2.4.B_7300651 | Zetaproteobacteria | 102 | 0.00 | Uncultured Mariprofundus (HQ206653) | 97.32 | Mariprofundus ferrooxydans (AATS01000006) | 96.42 |
| CHS.BG.2.4.B_1018757 | Zetaproteobacteria | 53 | 0.00 | Uncultured Mariprofundus (HQ206653) | 98.66 | Mariprofundus ferrooxydans (AATS01000006) | 97.77 |
| CHS.HV.0.2.B_6573067 | Zetaproteobacteria | 29 | 0.00 | Uncultured Mariprofundus (FJ905748) | 98.21 | Mariprofundus ferrooxydans (AATS01000006) | 96.88 |
| SOU.HV.16.18.B_1617388 | Deltaproteobacteria | 65 | 0.00 | Uncultured Desulfobacca (EU487900) | 95.1 | Dissulfurirhabdus thermomarina (KU051627) | 88.64 |
| SOU.HV.16.18.B_4079926 | Deltaproteobacteria | 50 | 0.00 | Uncultured Desulfobaccao (EU487900) | 95.1 | Dissulfurirhabdus thermomarina (KU051627) | 89.27 |
| SOU.HV.16.18.B_1885541 | Deltaproteobacteria | 47 | 0.00 | Uncultured Desulfobaccao (EU487900) | 95.1 | Dissulfurirhabdus thermomarina (KU051627) | 87.75 |
| SOU.HV.0.2.B_3891936 | Bacilli | 13250 | 0.63 | Planococcus maritimus (CP016538) | 98.43 | Planococcus maritimus (CP016538) | 98.43 |
| SOU.HV.0.2.B_5028604 | Bacilli | 9294 | 0.44 | Planococcus maritimus (CP016538) | 99.77 | Planococcus maritimus (CP016538) | 99.77 |
| SOU.HV.0.2.B_6185638 | Bacilli | 5669 | 0.27 | Planococcus maritimus (CP016538) | 99.33 | Planococcus maritimus (CP016538) | 99.33 |
| SOU.BG.0.2.B_4070160 | Gammaproteobacteria | 14290 | 0.68 | Pseudoalteromonas undina (X82140) | 95.98 | Pseudoalteromonas undina (X82140) | 95.98 |
| CHS.HV.0.2.B_1132728 | Gammaproteobacteria | 10326 | 0.49 | Pseudoalteromonas arabiensis (LRUF01000013) | 99.11 | Pseudoalteromonas arabiensis (LRUF01000013) | 99.11 |
| SOU.BG.0.2.B_4110066 | Gammaproteobacteria | 7298 | 0.35 | Pseudoalteromonas undina (X82140) | 99.32 | Pseudoalteromonas undina (X82140) | 99.32 |
| SOU.HV.8.10.B_5070135 | Gammaproteobacteria | 16274 | 0.77 | Pseudomonas zhaodongensis (JQ762275) | 99.55 | Pseudomonas zhaodongensis (JQ762275) | 99.55 |
| CHS.HV.16.18.B_7608234 | Gammaproteobacteria | 10130 | 0.48 | Pseudomonas songnenensis (JQ762269) | 98.88 | Pseudomonas songnenensis (JQ762269) | 98.88 |
| SOU.HV.8.10.B_5611531 | Gammaproteobacteria | 8039.00 | 0.38 | Pseudomonas zhaodongensis (JQ762275) | 99.07 | Pseudomonas zhaodongensis (JQ762275) | 99.07 |
| CHS.HV.16.18.B_4969249 | Alphaproteobacteria | 3506.00 | 0.17 | Paracoccus sediminis (JX126474) | 99.26 | Paracoccus sediminis (JX126474) | 99.26 |
| CHS.HV.16.18.B_4316628 | Alphaproteobacteria | 3257.00 | 0.15 | Paracoccus sediminis (JX126474) | 98.11 | Paracoccus sediminis (JX126474) | 98.11 |
| CHS.HV.16.18.B_86440 | Alphaproteobacteria | 2889.00 | 0.14 | Paracoccus sediminis (JX126474) | 99.51 | Paracoccus sediminis (JX126474) | 99.51 |
