# Supplementary material for: Bacterial Diversity and Biogeochemistry of Two Marine Shallow-Water Hydrothermal Systems off Dominica (Lesser Antilles)
Source: Front Microbiol. 2017 Dec 4;8:2400. doi: 10.3389/fmicb.2017.02400 (PMC5722836; doi:10.3389/fmicb.2017.02400)
Supplement: Supplementary file 5 [file Image1.PDF]

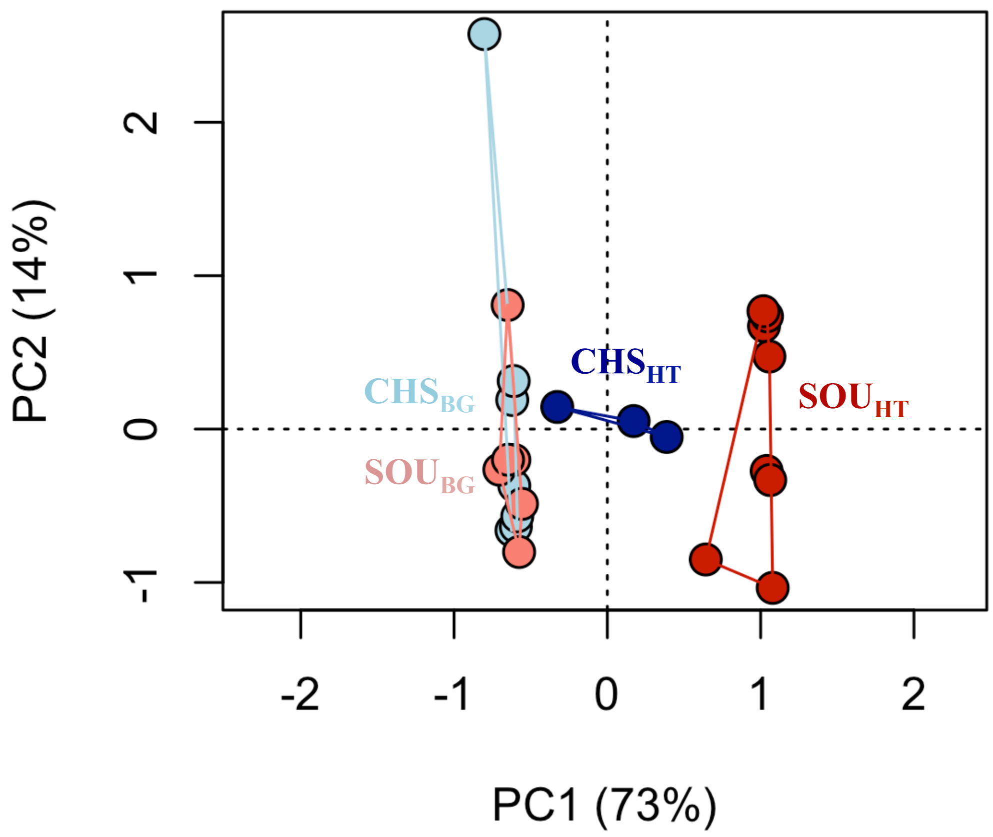

SUPPLEMENTARY FIGURE 1. Principal Component Analysis (PCA), revealing prominent differences in the porewater geochemistry between SOU$_{HT}$ and all other investigated sites. Samples are colour coded according to the sampling site.
