# Supplementary material for: Bacterial Diversity and Biogeochemistry of Two Marine Shallow-Water Hydrothermal Systems off Dominica (Lesser Antilles)
Source: Front Microbiol. 2017 Dec 4;8:2400. doi: 10.3389/fmicb.2017.02400 (PMC5722836; doi:10.3389/fmicb.2017.02400)
Supplement: Supplementary file 6 [file Image2.PDF]

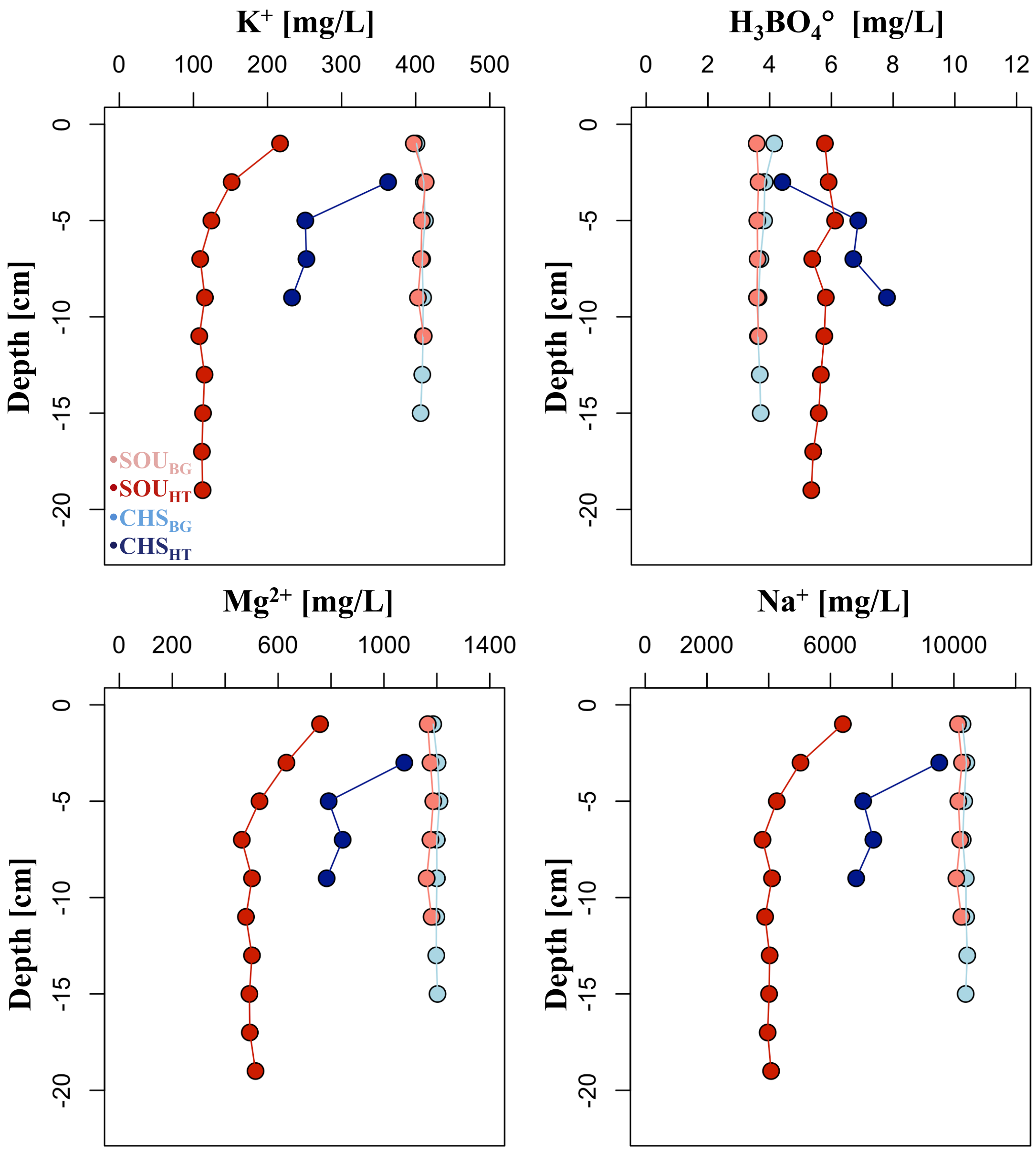

SUPPLEMENTARY FIGURE 2. Porewater geochemistry plots of potassium, boron, magnesium and sodium at the different sampling sites.
