# Supplementary material for: Bacterial Diversity and Biogeochemistry of Two Marine Shallow-Water Hydrothermal Systems off Dominica (Lesser Antilles)
Source: Front Microbiol. 2017 Dec 4;8:2400. doi: 10.3389/fmicb.2017.02400 (PMC5722836; doi:10.3389/fmicb.2017.02400)
Supplement: Supplementary file 7 [file Image3.PDF]

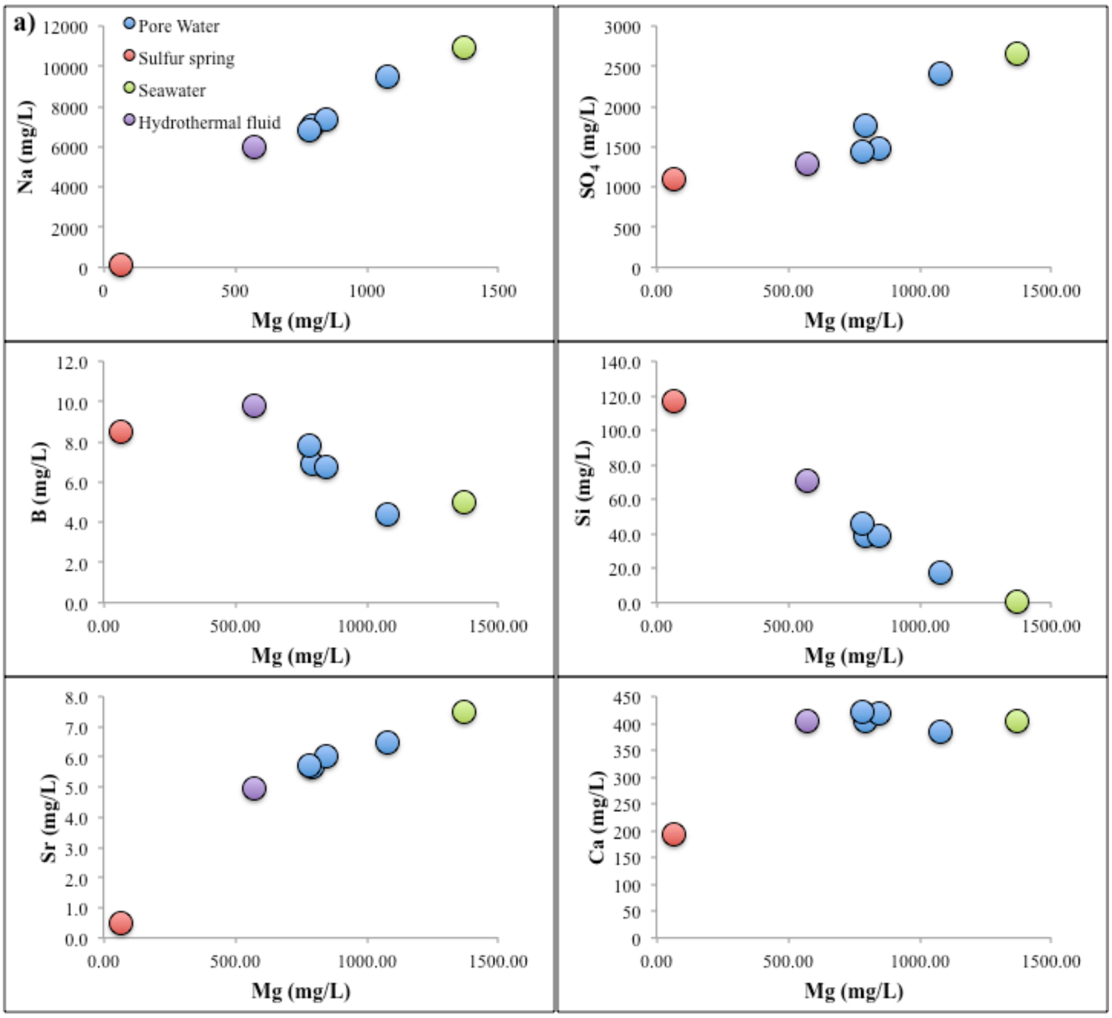

SUPPLEMENTARY FIGURE 3. Correlation between magnesium and other elements in the porewater from hydrothermal sites, hydrothermal fluid, hydrothermal end-member (Sulfur Spring) and seawater at (a) Champagne Hot Springs Bay (CHS_{HT}) and (b) Soufrière Bay (SOU_{HT}).

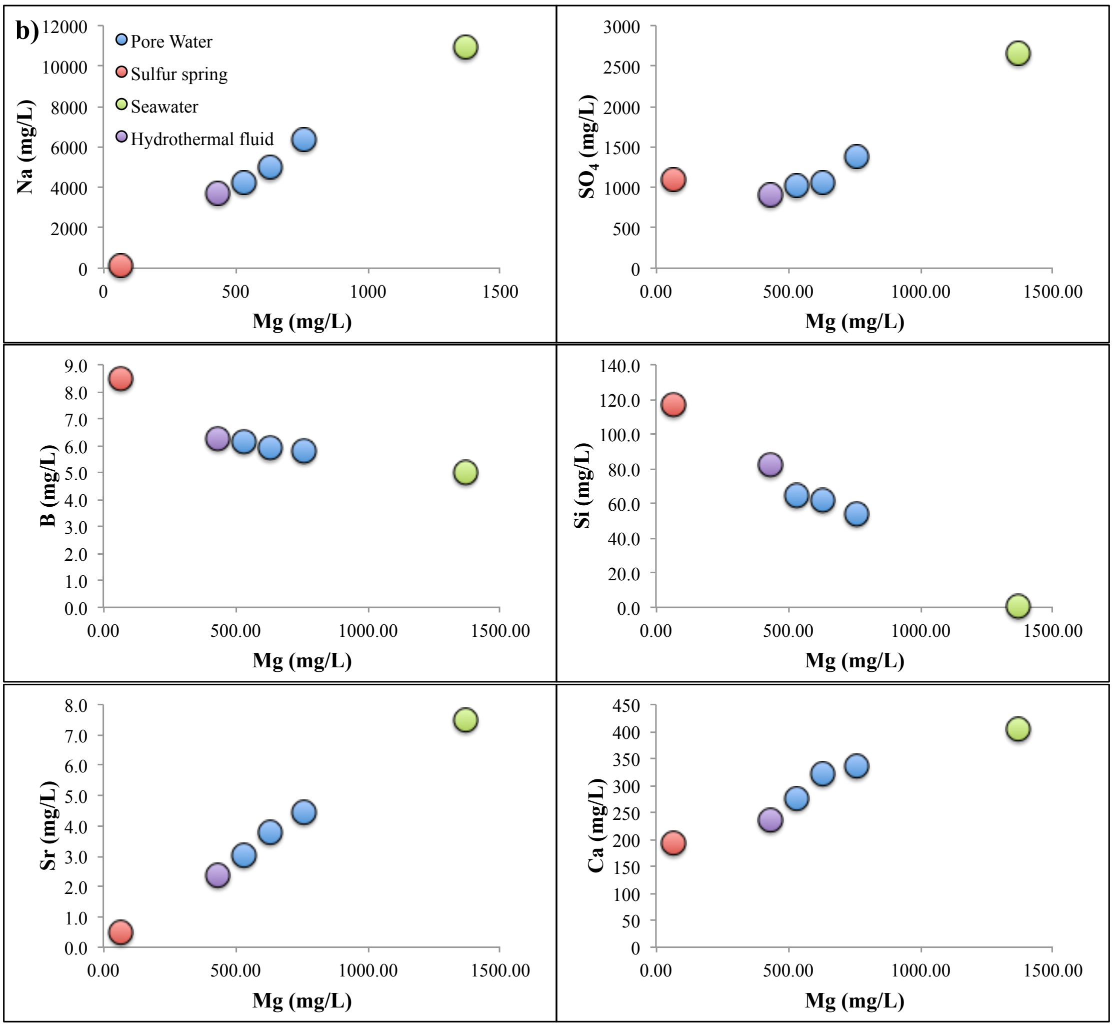
b)
Pore Water
Sulfur spring
Seawater
Hydrothermal fluid
Na (mg/L)
12000
10000
8000
6000
4000
2000
0
0
500
1000
1500
Mg (mg/L)
SO_4 (mg/L)
3000
2500
2000
1500
1000
500
0
0.00
500.00
1000.00
1500.00
Mg (mg/L)
B (mg/L)
9.0
8.0
7.0
6.0
5.0
4.0
3.0
2.0
1.0
0.0
0.00
500.00
1000.00
1500.00
Mg (mg/L)
Si (mg/L)
140.0
120.0
100.0
80.0
60.0
40.0
20.0
0.0
0.00
500.00
1000.00
1500.00
Mg (mg/L)
Sr (mg/L)
8.0
7.0
6.0
5.0
4.0
3.0
2.0
1.0
0.0
0.00
500.00
1000.00
1500.00
Mg (mg/L)
Ca (mg/L)
450
400
350
300
250
200
150
100
50
0
0.00
500.00
1000.00
1500.00
Mg (mg/L)
