# Supplementary figures and images for: Bacterial Diversity and Biogeochemistry of Two Marine Shallow-Water Hydrothermal Systems off Dominica (Lesser Antilles)
Source: Front Microbiol. 2017 Dec 4;8:2400. doi: 10.3389/fmicb.2017.02400 (PMC5722836; doi:10.3389/fmicb.2017.02400)

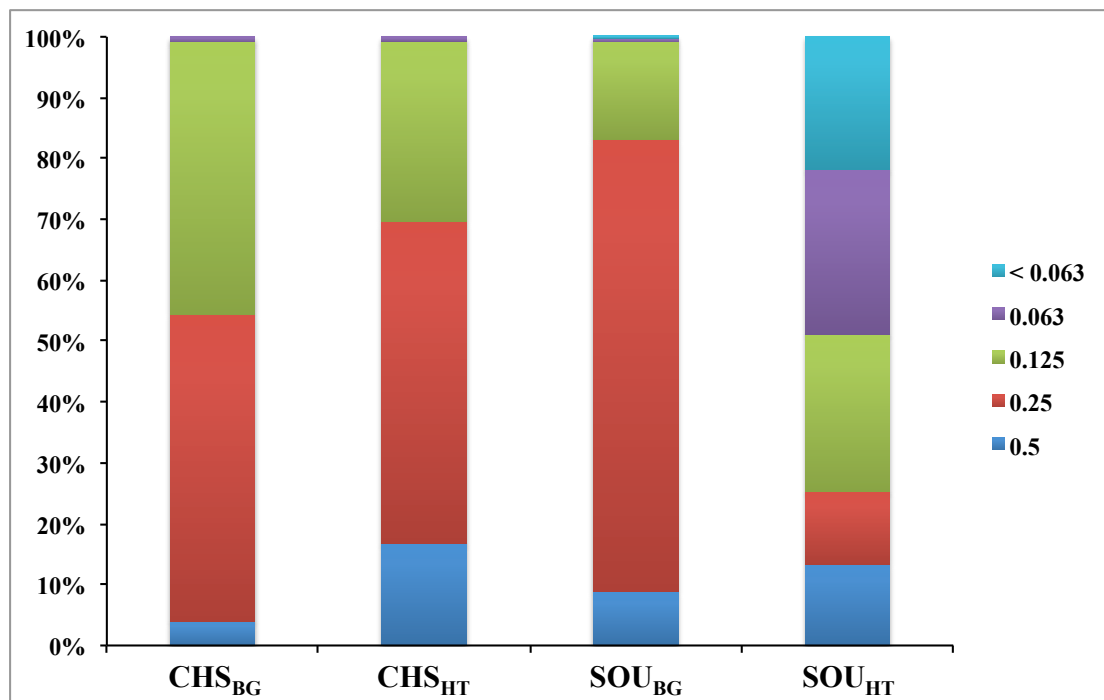

**SUPPLEMENTARY FIGURE 4. Size of sediment grains at all investigated sites at Dominica.**

Supplement: Supplementary file 8 [file Image4.PDF]
